# Single-cell RNA sequencing of batch Chlamydomonas cultures reveals heterogeneity in their diurnal cycle phase

**DOI:** 10.1093/plcell/koab025

**Published:** 2021-02-02

**Authors:** Feiyang Ma, Patrice A Salomé, Sabeeha S Merchant, Matteo Pellegrini

**Affiliations:** Department of Molecular, Cell and Developmental Biology, University of California, Los Angeles, California 90095, USA; Department of Chemistry and Biochemistry, University of California, Los Angeles, California 90095, USA; Institute for Genomics and Proteomics, University of California, Los Angeles, California 90095, USA; Department of Chemistry and Biochemistry, University of California, Los Angeles, California 90095, USA; Institute for Genomics and Proteomics, University of California, Los Angeles, California 90095, USA; Departments of Molecular and Cell Biology and Plant and Microbial Biology, University of California, Berkeley, California 94720, USA; Lawrence Berkeley National Laboratory, Berkeley, California 94720, USA; Department of Molecular, Cell and Developmental Biology, University of California, Los Angeles, California 90095, USA; Institute for Genomics and Proteomics, University of California, Los Angeles, California 90095, USA

## Abstract

The photosynthetic unicellular alga Chlamydomonas (*Chlamydomonas reinhardtii*) is a versatile reference for algal biology because of its ease of culture in the laboratory. Genomic and systems biology approaches have previously described transcriptome responses to environmental changes using bulk data, thus representing the average behavior from pools of cells. Here, we apply single-cell RNA sequencing (scRNA-seq) to probe the heterogeneity of Chlamydomonas cell populations under three environments and in two genotypes differing by the presence of a cell wall. First, we determined that RNA can be extracted from single algal cells with or without a cell wall, offering the possibility to sample natural algal communities. Second, scRNA-seq successfully separated single cells into nonoverlapping cell clusters according to their growth conditions. Cells exposed to iron or nitrogen deficiency were easily distinguished despite a shared tendency to arrest photosynthesis and cell division to economize resources. Notably, these groups of cells not only recapitulated known patterns observed with bulk RNA-seq but also revealed their inherent heterogeneity. A substantial source of variation between cells originated from their endogenous diurnal phase, although cultures were grown in constant light. We exploited this result to show that circadian iron responses may be conserved from algae to land plants. We document experimentally that bulk RNA-seq data represent an average of typically hidden heterogeneity in the population.

## Teaser

We show that single-cell RNA-seq (scRNA-seq) can be applied to Chlamydomonas cultures to reveal the heterogeneity in bulk cultures is largely driven by diurnal cycle phases)

## Introduction

Transcriptome analysis in the green unicellular alga Chlamydomonas (*Chlamydomonas reinhardtii*) has proliferated since the genome was released in 2007 (Merchant et al., 2007). Since then, dozens of experiments have been conducted that aimed to describe the changes in gene expression in response to changes in nutrient availability such as nitrogen (Plumley and Schmidt, 1989; Miller et al., 2010; Boyle et al., 2012; Blaby et al., 2013), sulfur (González-Ballester et al., 2010), phosphorus (Moseley et al., 2006; Schmollinger et al., 2014; Bajhaiya et al., 2016), acetate (Goodenough et al., 2014; Bogaert et al., 2019), and essential metals (Castruita et al., 2011; Blaby-Haas and Merchant, 2012; Urzica et al., 2012; Malasarn et al., 2013; Blaby-Haas et al., 2016), as well as changes that occur in response to light (Wakao et al., 2014; Tilbrook et al., 2016) or across the diurnal cycle (Zones et al., 2015; Strenkert et al., 2019), and following chemical treatments (Blaby et al., 2015; Wittkopp et al., 2017; Ma et al., 2020). A common feature of the prior studies is the use of bulk transcriptome deep sequencing (RNA-seq) obtained from the sequencing of RNA extracted from pools of cells. Such pooling is necessary to meet the material requirements for library preparation. Changes in transcript levels therefore reflect the average behavior of the culture and may not accurately inform on the extent of cell-to-cell variation that might exist in these samples.

Recently developed single-cell RNA sequencing (scRNA-seq) techniques have gained in popularity to counter the innate limitations of bulk RNA-seq. In Arabidopsis (*Arabidopsis thaliana*) and yeast (*Saccharomyces cerevisiae*), comparisons of bulk RNA-seq and scRNA-seq results have highlighted the heterogeneity of cell populations. For instance, the characterization of yeast culture responses to stress uncovered variability in gene expression between cells, which may shape how well they cope with the introduced stressor (Gasch et al., 2017). Individual yeast cells also do not age evenly within cultures, again highlighting the heterogeneity of bulk cultures (Zhang et al., 2020). Likewise, in Arabidopsis, profiling of single root cells revealed the stochasticity reflecting their developmental trajectories, although each cell type could be efficiently identified by comparing scRNA-seq and bulk RNA-seq data (Shulse et al., 2019; Zhang et al., 2019). In both Arabidopsis and yeast, the cells under investigation are surrounded by a physical barrier that must be removed prior to RNA extraction and library construction. In the case of Arabidopsis, the cell wall is digested by a mixture of enzymes for 60 min; protoplast isolation ahead of scRNA-seq may therefore introduce variation in the gene expression profile of single cells that must be considered during subsequent analysis, especially for short-lived RNAs.

In contrast to bulk RNA-seq data sets that inspect a few RNA samples to great depth (or coverage), scRNA-seq data sets provide an overview of the complement of genes that might be expressed in a given cell at very shallow coverage. Therefore, in a typical scRNA-seq data set, there are no values for most genes, either because they are truly not expressed or because their transcripts were not captured in that specific cell. Nevertheless, scRNA-seq data are also incredibly dense, as the data sets document the expression of *n* genes (usually thousands) across *m* cells (also in the hundreds to thousands), resulting in an *n × m* matrix whose gene expression data exist in an *n*-dimensional space. To at least partially remedy both limitations of scRNA-seq studies, dimensionality reduction methods are generally applied early on, bringing an estimate of the full data sets into a 2D space. Two popular methods presently in use are t-distributed Stochastic Neighbor Embedding (t-SNE) and Uniform Manifold Approximation and Projection (UMAP) (Van Der Maaten and Hinton, 2008; Becht et al., 2019). Both methods aim to preserve the local structure of complex data by converting the distance between neighboring cells into probabilities in *n*-dimensional space, followed by dimension reduction using different probability distribution functions and distance minimization parameters. Thus, genes with similar expression patterns will occupy similar *n*-dimensional neighborhoods that will be reduced to the same local 2D neighborhoods, even when some genes have missing data. Recent benchmarking of various dimensionality reduction techniques has illustrated the strengths and limitations of both methods: While they both correctly distinguish samples into large clusters, the relative distance and orientation of these clusters may not reflect the underlying structure. In addition, the exact nature of the analyzed samples also matters: Indeed, the clustering of cells that fall along a continuum such as a developmental time course more accurately represents the underlying structure than discrete samples, especially with UMAP (Heiser and Lau, 2020). Another useful tool in analyzing and visualizing single cell data is to aggregate the expression of multiple genes, selected based on prior knowledge of gene function or pathway. The combined expression across many cells will reinforce any potential observable signal, thus facilitating downstream analysis.

As a unicellular organism, Chlamydomonas presents an ideal system for the application of scRNA-seq to discover whether cultures exhibit similar stochasticity in their transcriptome as do Arabidopsis root cells, yeast, or mammalian cells. Although the alga can be easily synchronized to a 24-h cell division cycle by growth under light–dark cycles (12-h light/12-h dark), the vast majority of experimental conditions relies on cells grown in constant conditions. In addition, cultures need to be refreshed often so as to keep cells in an actively growing state. It is assumed that such cultures are globally asynchronous and represent a mixture of cells in various phases along the diurnal and cell cycles. However, this assumption has not been tested empirically.

We describe here the scRNA-seq analysis of gene expression for almost 60,000 cells derived from three growth conditions and two Chlamydomonas strains. We report that scRNA-seq successfully captures the same gene expression signatures as do bulk RNA-seq approaches. We further show that cells experiencing distinct growth conditions cluster independently from one another. Finally, we determine that bulk Chlamydomonas cultures grown in constant light are far from homogeneous and exhibit instead substantial variation in their diurnal cycle, although the distribution of these phases is not uniform. We then use the preferential diurnal phase exhibited by cells to demonstrate the likely conservation of circadian iron responses in Chlamydomonas, as diurnal phases are globally lagging in iron-deficient algal cells, as seen in Arabidopsis (Chen et al., 2013; Hong et al., 2013; Salomé et al., 2013).

## Results

### scRNA-seq of Chlamydomonas cells reflects their iron nutritional status

To determine whether scRNA-seq methodology is applicable off-the-shelf for profiling Chlamydomonas cultures, we tested the cell wall–deficient strain CC-5390 under two contrasting conditions: iron replete (Fe+), and iron deficient (Fe−). We grew a single culture for 3 d in constant light and in Fe+ conditions before splitting the culture into separate Fe+ and Fe− cultures. We measured cell density after 23 h and adjusted it to 1,200 cells mL^−1^ for Gel Bead in Emulsion (GEM) formation and single-cell library preparation. We reasoned that 1 d in the complete absence of Fe would be sufficient to induce a strong Fe deficiency response (Page et al., 2012) but would not be as drastic as prolonged Fe deficiency from the time of initial inoculation (Urzica et al., 2012). To test reproducibility, we also generated a third sample consisting of a mixture of the two samples at equal cell densities and proceeded with GEMs alongside the Fe+ and Fe− samples.

**Figure 1:**
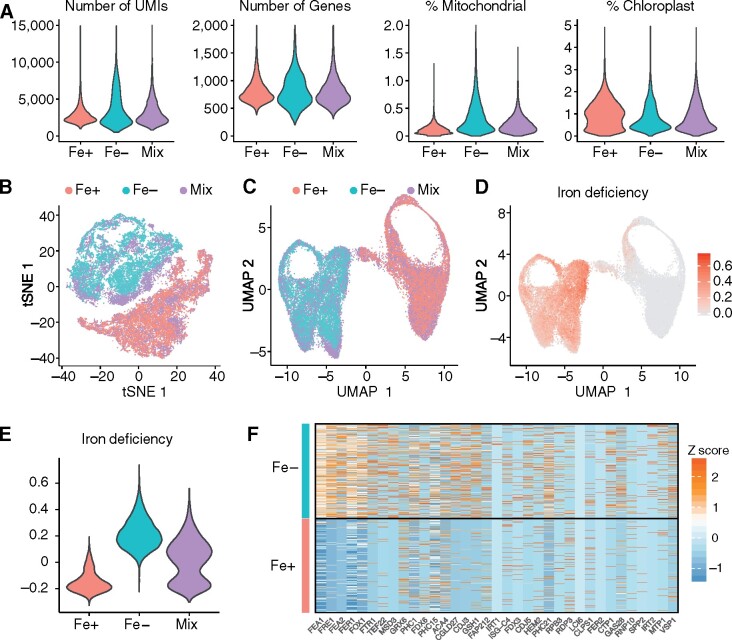
scRNA-seq properly separates chlamydomonas cells according to their iron nutritional status. In a first experiment, we grew Chlamydomonas strain CC-5390 in Fe-replete (Fe+) conditions before being transferred to Fe+ or Fe-limited conditions (Fe−) for 23 h. Cells were then processed for scRNA-seq, starting with GEMs formation in the 10X Genomics pipeline. See also Supplemental Tables 1 and 2. (A) Characteristics of sequencing results from Chromium Single Cell 3′ gene expression libraries (first experiment). Violin plots report the number of genes, number of UMIs, and the percentage of gene expression estimates coming from the mitochondrial and chloroplast organelles in Fe+ (pink), Fe− (teal), and an equal mix of cells from Fe+ and Fe− cultures (Mix, purple). (B, C) t-SNE (B) and UMAP (C) plot for the 28,690 sequenced cells, colored by sample: Fe+, pink; Fe−, teal; Mix: purple. Each dot represents one cell. (D) UMAP plot of the iron deficiency module score, which includes genes highly induced by Fe deficiency (Urzica et al., 2012). Dark red indicates individual cells with a high iron deficiency module score and thus in a Fe-limited nutritional state. (E) Iron deficiency module score for each sample, shown as violin plots. Fe+, pink; Fe−, teal; Mix: purple. Note the bimodal distribution of the Mix sample. Wilcoxon Rank Sum Test was performed between the Fe+ and Fe− cells, the p value was below 2.2 × 10^−16^. (F) Heatmap representation of normalized gene expression estimates for of genes induced under Fe deficiency in Fe+ and Fe− cells. Each horizontal line indicates the expression of the listed gene in one cell.

After sequencing and mapping reads to the Chlamydomonas reference genome (version v5.5), we counted 28,690 cells across the three samples, from which we detected an average of 3,344 unique molecular identifiers (UMIs) per cell mapping to an average 823 genes (Figure 1A;  Supplemental Tables 1 and 2). We identified transcripts from 16,982 distinct genes in at least one cell across all samples, with an individual gene being detected on average in 1,391 cells across the three samples (Supplemental Figure 1A and Supplemental Table 3). The contribution of mitochondrial and chloroplast transcripts to UMIs was low (0.23% for mitochondria and 0.91% for chloroplasts; Figure 1A), consistent with the initiation of reverse transcription from an oligo(dT) primer (Gallaher et al., 2018).

The scRNA-seq data set consisted of expression information from 16,982 genes across about 30,000 cells, such that the expression data are in a 16,982-dimension matrix. To visualize the data in two dimensions, we applied two widely used dimensionality reduction methods: t-SNE (Van Der Maaten and Hinton, 2008) and UMAP (McInnes et al., 2018; Becht et al., 2019), using the R package Seurat (Stuart et al., 2019). Both methods aim to preserve much of the local and global data structure, although UMAP has been proposed to perform better than t-SNE in representing complex data into a low-dimensional space (Becht et al., 2019; Heiser and Lau, 2020). Both methods also perform well on continuous data, such as a developmental time course that describes a cell type-specific progenitor and its gradual differentiation, with UMAP outperforming t-SNE (Heiser and Lau, 2020). Fe+ and Fe− cells formed two clearly separated groups with both methods, while the mixed cells sample was equally divided between the first two groups and closely overlapped with them in the t-SNE (Figure 1B) and UMAP plots (Figure 1C). Note that with both methods, the relative position of each cluster is not always informative, which is a known limitation of these dimensionality reduction methods. These results demonstrated that scRNA-seq 1) successfully separated cells according to their nutritional status (Fe-replete or Fe-deficient) and 2) had very good technical reproducibility between libraries processed in parallel, as evidenced by the overlap between the mixed cells samples and the two test groups.

To validate the observation that scRNA-seq captured the Fe nutritional status of our samples, we calculated an iron deficiency module score (Stuart et al., 2019) for each cell using genes induced under Fe deficiency previously identified using bulk RNA-seq (Urzica et al., 2012). A module score calculates the average expression of a given gene list, subtracted by the aggregated expression of randomly sampled control genes. A module score therefore partially circumvents the low coverage typical of scRNA-seq data by aggregating the expression of multiple genes of interest into a quantitative output that can be visualized either using the t-SNE and UMAP plots or as a violin plot representing the distribution of values across cells experiencing the same treatment. We discovered that Fe− cells exhibit a much higher iron deficiency module score compared to Fe+ cells, supporting the ability of scRNA-seq to capture expression differences resulting from distinct culture conditions (Figure 1, D and E). The mixed cells sample showed a bimodal distribution for the iron deficiency module score, in agreement with the equal contribution of Fe+ and Fe− cells (Figure 1E).

We also performed differential expression analysis between the Fe+ and Fe− cells and obtained 1,589 differentially expressed genes between these two conditions with a cutoff at adjusted *P* <0.05. Notably, 69 out of 100 genes induced by iron deficiency used in the module score calculation were differentially regulated. We also plotted the expression of a number of iron-related genes across all cells, shown as a heatmap in Figure 1F. We observed strong induction for genes encoding various components of the Fe assimilation machinery, such as the *Fe ASSIMILATORY* (*FEA*) genes *FEA1* and *FEA2*, the *FERRIC REDUCTASE FRE1*, the multicopper oxidase *FOX1*, and the Fe permease *FE TRANSPORTER* (*FTR1*). Other highly expressed genes across Fe− cells included *TEF22*, which is divergently transcribed from the same promoter sequences as *FEA1*; the low Fe-induced *MANGANESE SUPEROXIDE DISMUTASE 3* (*MSD3*); the Chloroplast DnaJ-like *CDJ3* and C*ONSERVED IN THE GREEN LINEAGE 27* (*CGLD27*) (Urzica et al., 2012). Likewise, the *COPPER TRANSPORTING P-type ATPase CTP1* was highly expressed only in Fe− cells. CTP1 is predicted to load Cu into FOX1 for full Fe deficiency responses (La Fontaine et al., 2002; Eriksson et al., 2004; Merchant et al., 2006). The high-affinity Fe transporter *IRT1* was seldom expressed in either Fe+ or Fe− cells, although the related transporter gene *IRT2* was induced in a large fraction of Fe− cells (Figure 1F). Finally, we noted high expression of a number of genes encoding cell wall-associated proteins: cell wall pherophorin-C (*PHC*) *PHC1* and *PHC21*, vegetative SP-rich *VSP1*, and *GAMETE-SPECIFIC 28* (*GAS28*) (Waffenschmidt et al., 1993; Rodriguez et al., 1999); and plasma membrane proteins such as autoinhibited Ca^2+^-ATPase 4 (*ACA4*), *METAL TRANSPORT PROTEIN1* (*MTP1*), and *LOW CO_2_-INDUCED 6* (*LCI6*). We interpret these highly induced genes as being part of the stress response of a Chlamydomonas strain lacking a cell wall.

scRNA-seq, therefore, efficiently captures comparable changes in the transcriptome relative to bulk RNA-seq when Chlamydomonas cells are grown in Fe+ and Fe− conditions.

### scRNA-seq recapitulates nitrogen deficiency bulk RNA sequencing signatures

**Figure 2.**
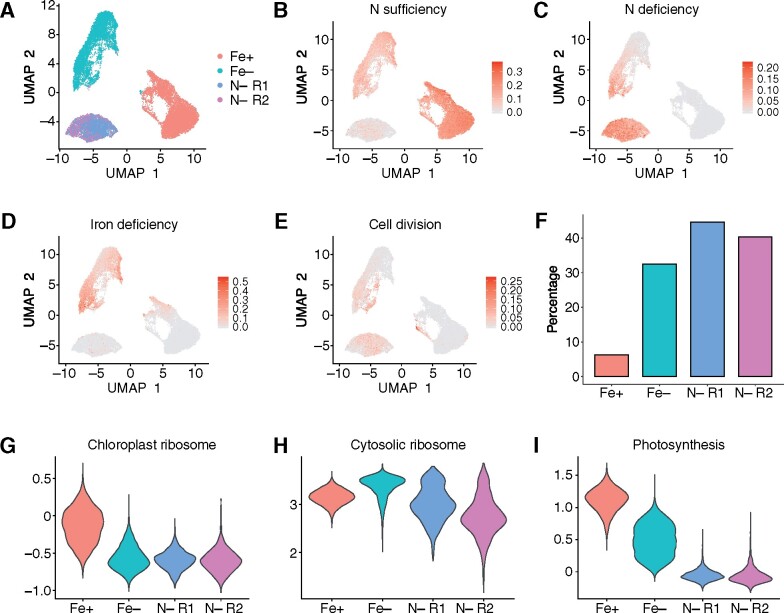
scRNA-seq captures bulk RNA sequencing signatures of nitrogen deficiency. We grew Chlamydomonas strain CC-5390 in nitrogen (N) and Fe-replete conditions before exposing cells to N deficiency (but full Fe supply) and Fe limitation (with full N supply) for 23 h. (A) UMAP plot for 19,140 sequenced cells, colored by sample: Fe+ and N+, red; Fe− and N+, teal; N− and Fe+, purple and magenta (two technical replicates: N− R1 and N− R2). (B) UMAP plot of the N sufficiency module score, which includes genes strongly repressed by N deficiency and/or induced by N sufficiency (Schmollinger et al., 2014). Dark red indicates individual cells with a high N sufficiency module score and are thus N-replete. (C) UMAP plot of the N deficiency module score, which includes genes highly induced by N deficiency (Schmollinger et al., 2014). Dark red indicates individual cells with a high N deficiency module score and thus in an N-limited nutritional state. (D) UMAP plot showing of iron deficiency module score, using the same gene list as in Figure 1. (E) UMAP plot showing of cell division module score, based on a list of genes involved in DNA replication and chromosome segregation with a mean diurnal phase of 12–14 h (using dawn as time 0). (F) Percentage of cells with a high cell division score across the Fe+, Fe−, and N− samples. We included cells with a positive cell division module score. (G–I), Module score across all samples for chloroplast *RPG*s (G), cytosolic ribosomes (H), and photosynthesis-related genes (I). The chloroplast and cytosolic *RPG* module score includes all nucleus-encoded plastid-localized or cytosolic RPG subunits, respectively. The photosynthesis module score is derived from all nucleus-encoded photosystem I and II components, as well as chlorophyll biosynthetic genes and M factors.

In a second independent experiment, we grew CC-5390 cells under replete conditions for both Fe and nitrogen (N) and then divided the cultures into Fe and N replete (control), Fe− (with full N supply) and N deficiency (N−, with full Fe supply, as technical duplicates) 23 h before processing cells for GEMs. After sequencing, we counted 19,140 cells across the four samples, from which we detected an average of 4,181 UMIs resolving into 694 genes per cell (Supplemental Figure 1B and Supplemental Tables 1–3). UMAP dimensionality reduction identified three clearly separated clusters, corresponding to replete cells (Fe+ N+), Fe-deficient cells (Fe− N+), and N-deficient cells (N− Fe+) (Figure 2A). These results indicated that scRNA-seq consistently produced distinct cell clusters for Fe+ and Fe− cells across multiple experiments (Figures 1, B and 2, A). In addition, N− cells formed a cluster that did not overlap with either Fe+ or Fe− cells, suggesting a transcriptome signature that is unique to each growth condition. Finally, we again observed good technical reproducibility, as the two replicates for N− cells closely overlapped.

To investigate whether scRNA-seq accurately captured the behavior of N status signature genes identified by bulk RNA-seq, we calculated module scores using two gene lists: genes repressed under N deficiency (and thus induced under N sufficiency conditions; Figure 2B) and genes induced under N deficiency (Figure 2C). Both Fe+ and Fe− cells showed a high N sufficiency module score, although Fe+ cells appeared to exhibit a higher score than Fe− cells (Figure 2B). In agreement, a subset of Fe− cells displayed a significant module score for N deficiency genes, as expected due to the rearrangement of the photosynthetic apparatus in response to Fe deficiency (Moseley et al., 2002). Notably, N− cells were characterized by a very low module score for N sufficiency marker genes and a high module score for N deficiency genes, thus validating their clustering into a group separate from those of Fe-replete and Fe-limited cells (Figure 2, B and C).

The Fe module score was high in Fe− cells, further confirming the UMAP clustering results (Figure 2D). As Fe− and N− cells would be predicted to stop dividing rapidly to maintain their nutritional quotas (Street and Paytan, 2005), we calculated a module score for genes specifically involved in cell division (minichromosome maintenance complex, DNA replication, and structural maintenance of chromosome-encoding genes). Overall, few cells showed a cell division signature, but they largely belonged to the Fe− and N− clusters (Figure 2D). We also observed a subgroup of Fe− cells with a strong cell division module score. We hypothesized that these highlighted cells were arrested prior to entry into cell division proper due to Fe or N deficiency. To test this hypothesis, we calculated the percentage of cells with a positive cell division module score for each sample: 30–40% of Fe− and N^−^ cells fulfilled this criterion, consistent with cell cycle arrest to prevent dilution of nutrients by division (Figure 2F). By contrast, only approximately 7% of Fe+ cells had a high cell division module score, as expected for an even distribution of cells along the various stages of the cell cycle. These results are consistent with a cell cycle block in nutrient-limited cells before cell division, as observed previously in Chlamydomonas cultures treated with cycloheximide (Howell et al., 1975).

Because of the high abundance of the photosynthetic apparatus, with a stoichiometry of 1 × 10^6^ molecules per cell, photosynthetic proteins constitute a high draw on the amino acid pool and on the Fe pool because of their high Fe content. Therefore, Fe and N deficiency are expected to have a strong negative effect on the biosynthesis of the photosynthetic apparatus, and especially in the case of N deficiency, the translation apparatus. We therefore calculated module scores for genes of the photosynthesis apparatus, as well as for ribosomal protein genes (*RPG*s). While mitochondrial *RPG*s showed a constant module score across all conditions (Supplemental Figure 2A), chloroplast *RPG*s were associated with a substantially reduced module score under Fe or N deficiency (Figure 2G). These results are consistent with the cellular response to each nutritional deficit: Fe deficiency will limit chloroplast development, while N deficiency will cause a global reallocation of N resources away from N-rich proteins such as ribosomes (Siersma and Chiang, 1971; Martin et al., 1976) or photosynthetic proteins (Plumley and Schmidt, 1989). This latter hypothesis was also reflected in the module score for cytosolic *RPG*s, which was much lower in N− cells relative to N+ cells (Figure 2H). Finally, the module score for photosynthetic genes recapitulated nicely the known physiological state of each group of cells, with Fe+ cells showing a high photosynthesis module score that decreased in Fe− cells (Figure 2I). N− cells experienced an even stronger repression of the photosynthetic apparatus, with a mean module score close to 0 (Figure 2I). These results independently confirmed the module scores calculated for N sufficiency and deficiency, as several genes encoding photosynthetic components (e.g. LIGHT-HARVESTING COMPLEX proteins 7 LHCAs and 4 LHCBs) are included in the N sufficiency list (Peltier and Schmidt, 1991; Moseley et al., 2002).

N deficiency is a routinely employed growth condition to induce the production of storage lipids from the remodeling of membrane lipids in Chlamydomonas. When we looked for genes involved in lipid biosynthesis, we detected no changes, as determined by a module score for lipid biosynthetic genes (Supplemental Figure 2B), as expected. However, our cultures experienced clear signs of N deficiency, as evidenced by severe chlorosis, suggesting that the increased expression of genes involved in triacylglyceride biosynthesis may be more delayed relative to N sparing mechanisms. To test this hypothesis, we looked at *PDAT1* (Cre02.g106400), *DGAT1* (Cre01.g045903), and *DGTT1* (Cre12.g557750): only *DGTT1* demonstrated a clear increase in expression in N− cells, while *PDAT1* and *DGAT1* did not (Supplemental Figure 2C). *DGTT1* was also more highly expressed than either gene in bulk RNA-seq experiments (Schmollinger et al., 2014), possibly hinting at the detection limit of scRNA-seq. We previously observed a 65% reduction in chlorophyll levels per cell within 24 h of transfer to N deficiency, concomitantly with a 50% decrease in total protein levels (Schmollinger et al., 2014). The same drop in chlorophyll levels was also reported in cultures grown in constant light and maintained for months in low N conditions (Plumley and Schmidt, 1989). Notably, cultures subjected to long-term low N fail to exhibit gametic activity (Plumley and Schmidt, 1989), although N deficiency is well-known to induce the gametic program (Martin and Goodenough, 1975). We therefore turned to a list of genes previously shown to be highly and specifically expressed in each gametic type (from *mt*^−^ and *mt*^+^ cells) (Lopez et al., 2015) and calculated the associated module score. As shown in Supplemental Figure 2, D and E, N-deficient cells showed a specific enrichment in *mt*^+-^specific genes, but not *mt*^−^-specific genes, relative to Fe-deficient cells and their sufficient control. This observation was in agreement with the genotype of the strain used here: CC-5390, which is of mating type *mt*^+^ (Strenkert et al., 2019). We also noted that the gametic module scores were fairly weak in terms of the magnitude of upregulation, but this may reflect the long 24-h N deficiency treatment used in this work. Indeed, the gametic transcriptional program is activated within 2–3 h of transfer into N depletion, and time points beyond 5–8 h are considered late-stage (Abe et al., 2004; Lopez et al., 2015). Notably, many of the gamete-specific genes identified by Lopez et al. showed no clear and/or sustained induction during N deficiency in either mating type over a 48-h time course in N deficiency conditions (Schmollinger et al., 2014), possibly contributing to the observed low module score. We did not test our N-deficient cultures for mating efficiency.

Together, these results demonstrate that scRNA-seq can sort individual cells according to their transcriptional profile in response to multiple stresses and that Fe− and N− cells are arrested before the completion of cell division, likely so as not to dilute their limiting resources and/or because they do not have the necessary resources to multiply.

### Diel rhythmic oscillations explain much of the heterogeneity of batch-cultured cells

**Figure 3.**
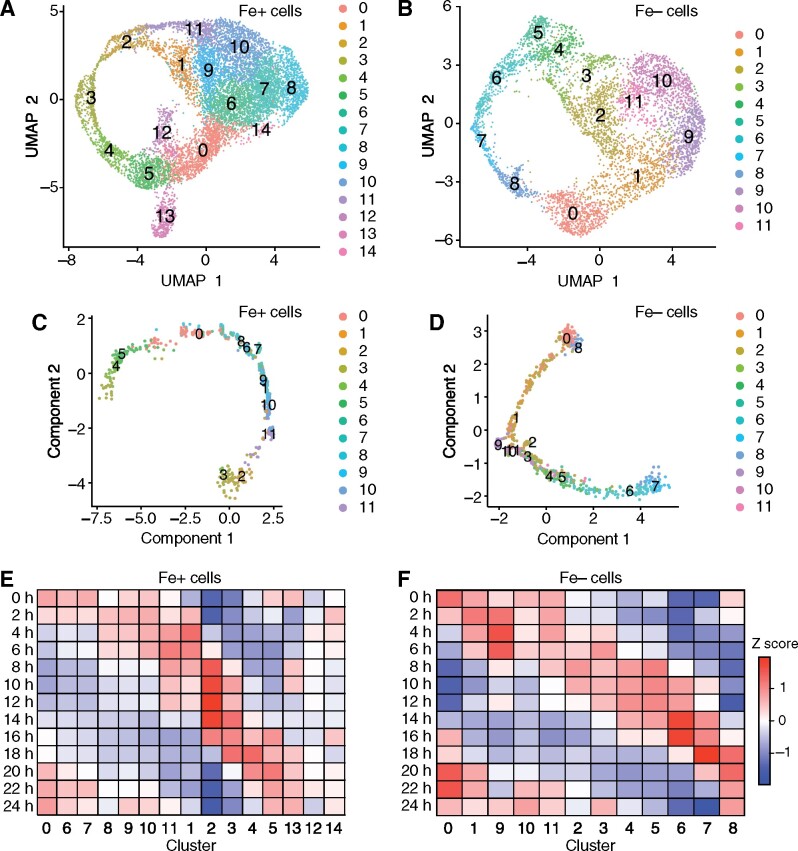
The endogenous diurnal phase of individual cells explains the heterogeneity of batch cell cultures. (A) UMAP plot for the 9,517 sequenced cells grown in Fe+ conditions from Experiment 1. The cells were separated into clusters by Seurat (Stuart et al., 2019) and are indicated by the color gradient, with the color key on the right side of the plot. (B) Same as (A), but with 9,748 sequenced cells grown in Fe− conditions from Experiment 1. (C, D), Trajectory plot of Fe+ (C) and Fe− (D) cells from Experiment 1, colored according to their constituent clusters, as determined by Monocle. (E, F), Heatmap representation of the average diurnal module scores associated with all clusters identified for Fe+ (E) and Fe− (F) cultures. We calculated a diurnal module score for each cluster in 1-h phase bins based on diurnal phase data reported by Zones et al. (2015) of high-confidence rhythmic genes, defined as the overlap of rhythmic genes from two recent studies (Zones et al., 2015; Strenkert et al., 2019).

One of the primary advantages of scRNA-seq is that it can reveal the heterogeneity between cells, while bulk RNA-seq only captures the average expression across all cells. In both experiments, we observed clear heterogeneity in both Fe^+^ and Fe− cells, as they occupied a rather large territory in both t-SNE and UMAP low-dimension projections. To explore the source of this heterogeneity in more detail, we applied the dimensionality reduction step only to the Fe+ cells, which were easily identifiable (Figure 1C). We then ran unsupervised clustering on the Fe+ cells from the first experiment using a K nearest neighbor algorithm, which identified 15 clusters (Figure 3A). Notably, many clusters organized around a closed circle on the UMAP, a pattern that was also present in the t-SNE plot, although not as pronounced (Supplemental Figure 3, A and B). UMAP was previously shown to provide meaningful organization of cell clusters, to preserve the global structure of the data and the continuity of the cell clusters, as might be expected of a developmental gradient across progenitors and terminally differentiated cells (McInnes et al., 2018; Heiser and Lau, 2020). Since our cultures are unlikely to differentiate, we hypothesized that the cells might have organized around the circle in a temporal fashion. We observed a similar circle in the second experiment (Figure 2A) and noted that a fraction of cells appeared to be primed for cell division based on the cell division module score (Figure 2, E and F). We also obtained 11 clusters with Fe-deficient cells that organized into a comparable circle (Figure 3B;  Supplemental Figure 3B), suggesting that such clustering may reflect a common behavior of Chlamydomonas cultures.

To determine whether the observed clusters might correlate with the endogenous diurnal phase of each cell, we first turned to a trajectory analysis with the R package Monocle (Trapnell et al., 2014). The principle of this analysis relies on the predictability of gene expression changes in cells undergoing a transition from state A to state B. Although this analysis is routinely applied to developmental data sets, it should be equally applicable to diurnal and circadian data with predictable gene expression changes over the diurnal or circadian cycle. Monocle identified a single trajectory for both Fe-sufficient and Fe-deficient cells (Figure 3, C and D; Supplemental Figure 4, A–D) and allowed a clear ordering of clusters. Satisfyingly, clusters #1 and #6–11 grouped closely together in the trajectory obtained for Fe+ cells, although these clusters covered a large area of the UMAP plot, suggesting a shared expression signature. Likewise, clusters #1–3 and #9–11 concentrated in the same portion of the trajectory deduced by Monocle. The identification of a single trajectory devoid of any side branches also suggests that Chlamydomonas cultures occupy a continuum of possible states along a single variable, in this case: Time.

**Figure 4.**
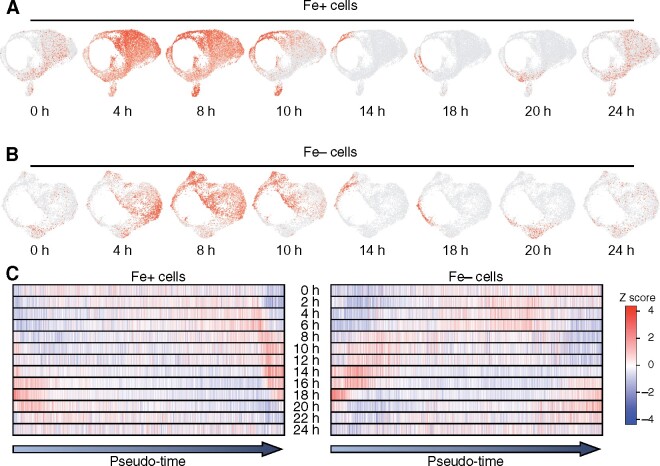
Pseudo-time construction aligns Fe+ cells along the diurnal cycle. (A) UMAP plots of representative diurnal module scores for Fe+ cells from the first experiment. (B) UMAP plots of representative diurnal module scores for Fe− cells from the first experiment. (C) Heatmap representation of the diurnal module score in individual cells, ordered by their pseudo-time, as determined by Monocle. Each vertical bar corresponds to one individual cell.

We next used the diurnal phases reported for Chlamydomonas cultures from two recent diurnal time-courses (Zones et al., 2015; Strenkert et al., 2019). We calculated a module score for rhythmic genes in 1-h time bins every other h, from 0 h to 24 h, for all clusters. The module scores were converted to a heatmap for ease of comparison and ordered according to the order deduced from the Monocle trajectory. As shown in Figure 3E, the resulting phase module scores followed a clear pattern that ordered the clusters along the diurnal cycle, with cluster #0 exhibiting a phase close to dawn and clusters #2 and #3 showing a phase close to dusk. Fe-deficient cells broadly followed a similar pattern (Figure 3F). We also plotted representative module scores in UMAP plots (Figure 4, A and B). Most cells occupied time bins between 4 h and 8 h after lights on. Smaller cell populations had time signatures closer to 14 h after dawn (largely overlapping with cluster #2), 18 h (corresponding to clusters #3 and #4), and 20 h (matching clusters #5 and #0). As expected for cells progressing through a ∼24-h rhythm, module scores for the phase bins at 0 and 24 h were very similar in our analysis (Figure 4A).

Plotting phase module scores in UMAP plots also provided an opportunity to compare the phase distribution of Fe+ and Fe− cells. Indeed, even though we collected cells at a single time point, phase module scores reveal the endogenous phase of each cell, as a molecular timetable analysis would (Ueda et al., 2004). When we plotted diurnal module scores in UMAP plots for Fe− cells, we observed a similar pattern as that seen with Fe+ cells (Figure 4B). However, we discovered through a careful inspection of the UMAP plots that Fe− cells appeared to display a later diurnal phase relative to Fe+ cells, with more Fe− cells represented in the 8-h phase module plots, while Fe+ cells were more numerous in the 4-h and 8-h modules (Figure 4, A and B). We interpret these results as suggestive of a delay in the circadian clock of the alga, reminiscent of the period lengthening effects observed under poor Fe nutrition in Arabidopsis (Chen et al., 2013; Hong et al., 2013; Salomé et al., 2013).

### Pseudo-time construction reveals the phase ordering of batch cultures

Until this point, we have considered one cell cluster as a unit and projected the diurnal module scores onto the clusters (Figure 3). To better characterize the rhythmic status of single cells, we ordered all cells on the basis of their individual trajectory time (also called pseudo-time), as determined by Monocle and illustrated in Supplemental Figure 4E. We ordered the cells into a continuous trajectory and assigned a pseudo-time to each cell. Next, we ordered cells by their pseudo-time and plotted their associated diurnal module scores (Figure 4C). The pseudo-time trajectory started with cells from clusters #4 to #5 for Fe+ cells, with a strong 18-h signature, that is shortly after cell division has occurred (Supplemental Figure 5, A and B). As pseudo-time increased, the trajectory progressed from cluster #0 through all other clusters in a counterclockwise fashion, to end with clusters #2 and #3, with a strong time signature around 14 h that corresponds to cell division (Supplemental Figure 5B). Pseudo-time analysis placed cell division in Fe-deficient cells around pseudo-time 3, consistent with a delay in cell division relative to Fe-replete cells. The remaining population of Fe− cells did not exhibit a high cell division module score along the pseudo-time trajectory. We interpret this result as another indication that Fe-deficient cells stop dividing to hold on to their remaining iron stores and not fall below a minimum iron quota (Supplemental Figure 5, C and D).

That pseudo-time analysis tracked the diurnal phase bins underscores the essential contribution of rhythmic gene expression to the heterogeneity of Chlamydomonas cells in batch cultures.

### Effects of the cell wall on RNA extractability and quality for scRNA-seq

**Figure 5.**
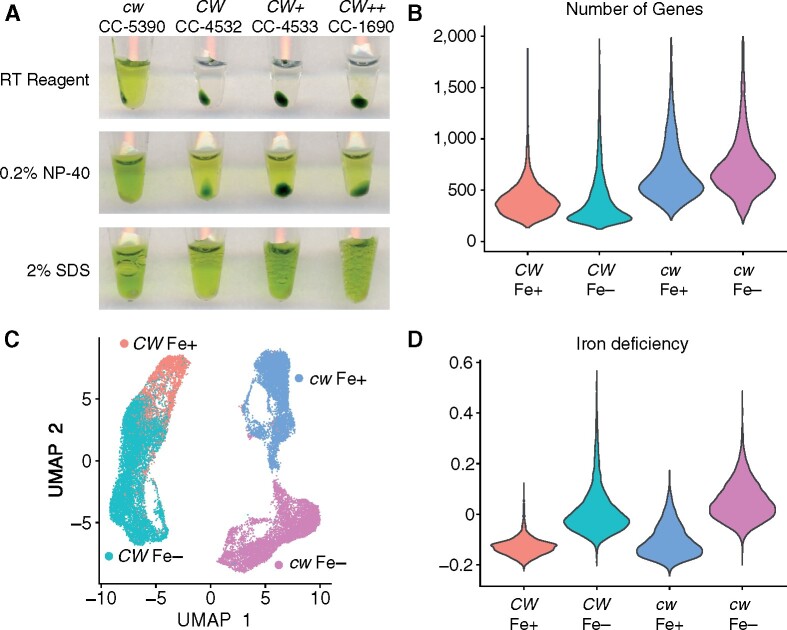
The chlamydomonas cell wall does not block RNA extraction for scRNA-seq analysis. (A) Testing cell lysis with the RNA extraction buffer included in the 10X Chromium pipeline. We grew strains without (CC-5390, *cw*) or with (CC-4532, CC-4533, CC-1690, *CW*) a cell wall for 3 d in TAP medium before taking a 100-µL aliquot. After collection by centrifugation, cells were incubated with RT reagent (10X Genomics), 0.2% NP-40 or 2% SDS and incubated for 15 min before spinning cells again and taking the photograph. Strain CC-1690 has a thicker cell wall than CC-4532, as indicated by “++.” (B) Number of genes from which UMIs were detected in each sample. Strain CC-4532 was grown alongside strain CC-5390 during Experiment 2 and treated in an identical manner. (C) UMAP plot of 24,795 sequenced cells from Experiment 2, using Fe status and the presence of the cell wall as variables. (D) Iron deficiency module score associated with the cells shown in (C). For (B-D) Red: CC-4532 (*CW*) Fe+; teal: CC-4532 (*CW*) Fe−; blue: CC-5390 (*cw*) Fe+; magenta: CC-5390 (*cw*) Fe−.

Protocols for the extraction of high-quality total RNA from Chlamydomonas cultures have been optimized to quickly inactivate ribonucleases that might be released from other cellular compartments during the thawing of a frozen cell pellet. For example, our routine RNA extraction protocol relies on the resuspension of the cell pellet in 2% sodium dodecyl sulfate (SDS) and proteinase K immediately after collection and prior to flash-freezing, conditions that are much harsher than the typical extraction procedures used in the 10X pipeline. Therefore, we first used a *cw* mutant of Chlamydomonas for the previous analyses to facilitate RNA extraction and recovery. However, to apply these methods to natural field conditions or commercial pond situations, it would be useful to understand whether the same methodology might apply to walled cells. As a preliminary test, we incubated Chlamydomonas cells from strains with or without cell wall in the RNA extraction buffer used in the early steps before library construction. We also treated equal numbers of cells with 0.2% NP-40 and 2% SDS as positive controls for cell lysis, as judged by the release of chlorophyll from the cell pellet. As shown in Figure 5A, only the strain CC-5390, which lacks a cell wall, resulted in substantial lysis in the RT kit buffer, while we failed to observe signs of lysis with the other cell wall-containing strains CC-4532, CC4533, and CC-1690.

Nevertheless, we selected strain CC-4532 (*CW*) for scRNA-seq on cells grown under iron-replete (Fe+) or Fe-starved (Fe−) conditions following the same methodology as for CC-5390. We processed both samples for GEMs production and library preparation. We successfully recovered RNA suitable for sequencing from these samples corresponding to 2,814 Fe+ cells and 9,289 Fe− cells. When compared to CC-5390 (*cw*) strain grown under the same conditions, we collected data from fewer genes, reflecting some differences in RNA extractability or UMI formation in strains without (*cw*) or with (*CW*) a cell wall (Figure 5B).

To determine whether scRNA-seq captured the Fe nutritional status of strain CC-4532, we performed UMAP dimensionality reduction on CC-5390 (*cw*) and CC4532 (*CW*) samples grown side by side and treated in an identical manner as part of the second experiment. First, we noticed that the two strains clustered separately from each other, indicating strong transcriptomic differences correlated with the absence of the cell wall, strain-specific differences (Gallaher et al., 2015) or both (Figure 5C). Both strains formed distinct clusters corresponding to Fe+ and Fe− cells, demonstrating the applicability of scRNA-seq analysis to cell wall-containing algal strains, even without resorting to mechanical or enzymatic digestion. We noticed that the cluster formed by CC-4532 Fe− cells overlapped with that of CC-4532 Fe+ cells (Figure 5C). The Fe module score supported this observation (Figure 5D). We hypothesize that transferring cells from Fe-replete to Fe-starved conditions for 23 h was sufficient to induce a strong Fe deficiency response in CC-5390, whereas the cell wall–containing strain CC-4532 only partially depleted its Fe stores. Although this hypothesis has never been tested in two isogenic Chlamydomonas strains only differing at the *CW15* locus, empirical phenotyping of strains with and without cell walls under low Fe conditions is consistent with the higher sensitivity of *cw* strains to Fe deficiency (Allen et al., 2007; Gallaher et al., 2015).

## Discussion

We show that scRNA-seq can recapitulate bulk RNA-seq signatures and separate individual cells in nonoverlapping clusters reflective of the growth condition they experienced (here, nutritional deficiency for Fe or N). In addition, we determine that Chlamydomonas cells grown in batch cultures retain substantial rhythmicity even after growing in constant light for weeks, contrary to common belief. This strong rhythmic component can explain much of the heterogeneity exhibited by individual Chlamydomonas cells in their transcriptional profile, as previously noted (Damodaran et al., 2015).

Using Arabidopsis and hairy bittercress (*Cardamine hirsuta*) as model systems, we had previously established that the Arabidopsis circadian clock responded to available Fe supply (Salomé et al., 2013). We and others showed that the circadian period lengthens under conditions of poor Fe nutrition, a phenotype that depended entirely on light-mediated chloroplast development (Chen et al., 2013; Hong et al., 2013; Salomé et al., 2013). One of several outstanding questions concerned the degree of evolutionary conservation of this response: do green single-cell algae such as Chlamydomonas adjust the period or phase of their circadian clock to the Fe status surrounding them? The comparison of diurnal phase module scores between Fe+ and Fe− Chlamydomonas cultures indicates that, in fact, Chlamydomonas cells do appear to adjust their diurnal phase as a function of their Fe status (Figures 3 and 4). In addition, they do so in the same direction as do Arabidopsis and hairy bittercress, with a delay in diurnal phase under poor Fe nutrition conditions. Although our growth conditions did not specifically control for circadian behavior, these results nonetheless tentatively suggest that circadian Fe responses may be conserved between Chlamydomonas and Arabidopsis, opening new avenues for the systematic dissection of the underlying molecular mechanism by looking for conserved genes shared by the alga and the land plant.

Our Chlamydomonas cultures were maintained in a constant light for weeks before sample collection. Yet, they showed a remarkable degree of synchronization that was not entirely expected. However, we independently reached the same conclusion from a deep reanalysis of hundreds of RNA-seq samples collected by our laboratory and the Chlamydomonas community over the past 10 years (Salomé and Merchant, 2021). Notably, one-third of all bulk RNA-seq samples showed the same preferred diurnal phase as the single cell data described here. We hypothesize that Chlamydomonas cells may remain synchronized over such periods of time through two (nonmutually exclusive) hypotheses: (1) growing cells establish a population-wide phase, similar to quorum sensing in bacteria, that would maintain them in a synchronized state to share resources and (2) the manipulation of cells, for example the inoculation of the test cultures, acts as a synchronizing signal that persists for days. This latter possibility would be similar to a nutritional synchronization, such as serum shocks applied to mammalian cell cultures (Balsalobre et al., 1998). Cultures grown in flasks demand serial dilutions to remain in their exponential growth phase, making it difficult to determine the contribution of dilution to synchronization. By contrast, continuous-flow bioreactors allow for absolute control of all parameters during cell culture, including cell density. We therefore envisage that the effect from inoculation as a resetting signal may be testable in bioreactors, whereby Chlamydomonas cells would be entrained by light–dark cycles and then released into constant light, all the while keeping the cell density low and constant. Samples may be collected every 12–24 h and processed for scRNA-seq, and the rhythmic components extracted as we did here, essentially following a molecular timetable approach applied to single cell populations (Ueda et al., 2004).

Our results also have commercial and ecological applications. Indeed, algal cells grown in large cultivation ponds may experience their surrounding environment differently as a function of pond depth, volume, cell density, and turbulence. While bulk RNA-seq may help determine the average molecular and physiological phenotypes of cells collected at various depths and positions within the pond, the inherent variation between cells will be lost. By contrast, scRNA-seq offers a much more detailed picture of all cells within each sample, thus raising sensitivity by several orders of magnitude. Likewise, scRNA-seq applied to environmental samples collected in the wild may make it possible to describe algae in their native environment—what stresses they may experience and their interactions with other organisms with which they share the same ecological niche. Our results demonstrate that although cells lacking sufficient Fe or N stall along the cell cycle (Figure 2), they also express key stress marker genes that are inherently specific for each stress they may encounter. With carefully formulated gene lists and the calculation of the corresponding module scores, scRNA-seq may thus provide a unique opportunity to study Chlamydomonas (and other algae) in the wild.

Chlamydomonas cells, just like yeast cells, can present a significant cell wall that might be considered a physical barrier for RNA extraction from single cells. In yeast, this technical limitation was resolved by adding the cell wall-digesting enzyme zymolyase before (Jackson et al., 2020) or during (Jariani et al., 2020) the reverse transcription step of the same 10X Chromium Single Cell 30 v2 protocol we followed here. However, it should be noted that the authors did not attempt to generate scRNA-seq libraries from walled (undigested) yeast cells. Using chlorophyll release as a proxy for cell lysis, we similarly saw little lysis for the walled strain CC-4532; nevertheless, we detected hundreds of UMIs from this strain, indicating that Chlamydomonas strains of various cell wall thicknesses may be amenable to scRNA-seq. The Chlamydomonas cell wall is composed of a mixture of proteins and glycoproteins arranged in multiple layers, potentially limiting the use of cell wall-digesting enzymes. A classic approach for the removal of the cell wall relies on autolysin, a zinc metalloprotease that is secreted by gametes during the initial stages of the algal sexual cycle. However, treating cells with autolysin may also induce the expression of the gametic program, as shown with the gamete-specific (*GAS*) genes *GAS28*, *GAS29*, and *GAS30*, even with a short incubation time of 30 min (Hoffmann and Beck, 2005). Another potential limitation to the use of autolysin is the difficulty associated with its purification from mating cells. A commercially available protease would thus be preferable, such as alcalase, a commercial form of subtilisin that shows 35% identity with sporangin, the so-called hatching enzyme responsible for the digestion of the cell wall surrounding daughter cells before their release (Kubo et al., 2009; Hwang et al., 2019).

We only tested scRNA-seq on strains with no (like CC-5390) or moderately thick (like C-4532) cell wall. However, other laboratories focus on strains with a much more developed cell wall, for example CC-4533 (the wild-type background for a large insertional mutant library, Li et al., 2019) and CC-1690. The microfluidics pipeline from 10X Genomics now provides the perfect basis for a systematic comparison of RNA extraction efficiency across Chlamydomonas strains, with or without the addition of a protease during the reverse transcription step. The information gathered will also directly apply to wild isolates with walls, since the *cw* strains were all generated by mutagenesis in the laboratory (Hyams and Davies, 1972). Finally, our results can provide a benchmark for comparing the recovery of RNA suitable for sequencing from various methods to preserve cells between sample collection and processing, such as freezing with or without the use of sample preservative solutions.

In conclusion, we showed that scRNA-seq can be applied to Chlamydomonas strains with or without a cell wall. In addition, scRNA-seq results recapitulated bulk RNA-seq data, indicating their reliability and the robustness of the Chlamydomonas transcriptome response to changes in its environment. Finally, we demonstrated that Chlamydomonas cells occupied a range of diurnal phases that may explain the heterogeneity exhibited by individual cells in bulk culture mode. By extracting diurnal data from single time point scRNA-seq, we also observed a delay in the phase of the Chlamydomonas diurnal clock, suggesting that, just like land plants, algae may adjust the pace of their rhythms to Fe availability. The application of scRNA-seq to cultivation ponds and natural isolates will pave the way to a deeper understanding of the interactions between algae and their surroundings.

## Materials and methods

### Growth conditions

We used the *C. reinhardtii* strains CC-5390 (*cw15 arg7-8::ARG7 mt^+^*) and CC-4532 (*CW mt^−^*), which we procured from laboratory stocks. We grew all pre-cultures in Tris Acetate Phosphate (TAP) medium supplemented with micronutrients as described previously (Kropat et al., 2011), at 24°C in constant light (provided by a mixture of cool-white and warm-white fluorescent light bulbs, for a total Photon Flux Density ∼50 µmol m^−2^ s^−1^) and under constant agitation (180 rpm) in an Innova-44R incubator.

In the first experiment, we started a pre-culture of strain CC-5390 in 50 mL TAP medium with 20 µM FeEDTA (iron-replete conditions) at an initial cell density of 5 × 10^4^ cells mL^−1^. After 5 d, we inoculated a new pre-culture at the same initial cell density (5 × 10^4^ cells mL^−1^), with 100 mL TAP medium + 20 µM FeEDTA in a 250-mL flask. After another 5 d, we collected the cells by centrifugation for 3 min at 1,600*g* at room temperature using an Eppendorf centrifuge (model 5810 R), resuspended the pellet in 10 mL of fresh TAP medium (with 20 µM FeEDTA), and used 1 mL to inoculate a fresh flask containing 100 mL TAP medium + 20 µM FeEDTA, resulting in a 10-fold dilution of the culture. The next day, we pelleted the culture again across two 50-mL Falcon tubes, washed the pellets once with TAP medium without FeEDTA, and resuspended each pellet with either 50-mL TAP medium without FeEDTA (Fe− condition) or with 50-mL TAP medium + 20 µM FeEDTA (Fe+ condition) before transferring the test cultures into fresh sterile 250-mL flasks and placing the flasks into the incubator. After 23 h of growth, we counted cell density in both cultures on a hemocytometer. Target cell density for scRNA-seq analysis is 1,200 cells µL^−1^: we therefore transferred 1.2 × 10^6^ cells mL^−1^ in a 1.5-mL Eppendorf tube, centrifuged the cells briefly on a tabletop centrifuge at 400*g* at room temperature. We resuspended the pellets into 1× phosphate-buffered saline (PBS) with 0.04% bovine serum albumin (BSA), placed the tubes on ice, and covered them with aluminum foil. We walked to the Technology Center for Genomics and Bioinformatics at UCLA Pathology and Medicine (∼5 min) for immediate processing, starting with GEMs formation.

For the second experiment, we started pre-cultures for CC-4532 and CC-5390 in 50-mL TAP medium + 20 µM FeEDTA at an initial cell density of 5 × 10^4^ cells mL^−1^. After 3 d, we inoculated a new culture at the same initial cell density (four flasks for CC-5390 and two flasks for CC-4532). After another 3 d, we refreshed the cultures by 1:2 dilution with fresh TAP medium + 20 µM FeEDTA. The next day, we resuspended cultures in TAP without FeEDTA, TAP + 20 µM FeEDTA or TAP – nitrogen (CC-5390) or in TAP without FeEDTA or TAP + 20 µM FeEDTA (CC-4532), as described above. After 23 h of growth, we counted cells and proceeded as above.

### 10X library preparation, sequencing, and alignment

Cells were washed with PBS with 0.04% BSA, then counted with Countess II automated Cell Counter (Thermo Fisher, Waltham, MA). We loaded 10,000 cells onto the 10X Chromium Controller using Chromium Single Cell 3′ gene expression reagents (10X Genomics, Pleasanton, CA). The sequencing libraries were prepared following the manufacturer’s instructions (10X Genomics), with 12 cycles used for cDNA amplification and 12 cycles for library amplification. Library concentrations and quality were measured using Qubit ds DNA HS Assay kit (Life Technologies, Carlsbad, CA) and Agilent Tapestation 4200 (Agilent, Santa Clara, CA). The libraries were sequenced on a NextSeq500 platform as 2 × 50 paired-end reads to a depth of approximately 150 million reads per library (Experiment 1), or using 2 × 50 paired-end reads, on an Illumina NovaSeq 6000 S2 platform to a depth of approximately 300 million reads per library (Experiment 2). Raw reads were aligned to the Chlamydomonas genome (*C. reinhardtii* v5.5, Blaby et al., 2014) and cells were called using cellranger count (v3.0.2, 10X Genomics). Individual samples were aggregated to generate the merged digital expression matrix using the cellranger aggr pipeline (10X Genomics). 

### scRNA-seq data analysis

The R package Seurat (v3.1.2) (Stuart et al., 2019) was used to cluster the cells in the digital expression matrix. We filtered out cells with fewer than 100 genes or 300 UMIs detected as low-quality cells. We divided the gene counts for each cell by the total gene counts for that cell, multiplied by a scale factor of 10,000, then natural-log transformed the counts. We used the FindVariableFeatures function from Seurat to select variable genes with default parameters. We used the ScaleData function from Seurat to scale and center the counts in the data set. We performed principal component analysis on the variable genes and selected 20 principle components for cell clustering (resolution = 0.5) and UMAP dimensionality reduction. We clustered the cells using a K-nearest neighbor method, which assesses which K value results in the smallest between-cell distance within and between clusters. The cells were embedded in a K-nearest neighbor graph, with edges drawn between cells with similar expression patterns. The cells were then partitioned into highly interconnected clusters. We calculated module scores using the AddModuleScore function with default parameters. A module score calculates the average expression of a given gene list, subtracted by the aggregated expression of randomly sampled control genes. To calculate differentially expressed genes, the Wilcoxon rank sum test was conducted, and the Benjamini–Hochberg Procedure was applied to adjust the false discovery rate. We considered genes with adjusted *P* <0.05 as significantly differentially expressed.

### Calculation of the diurnal module scores

We generated a list of diurnal signature genes by determining the overlap between rhythmic genes from two recent studies (Zones et al., 2015; Strenkert et al., 2019). The list contains 50 time points ranging from 0 h to 24.5 h in 30 min interval. To calculate module scores from nonoverlapping diurnal gene lists, we selected a three time point interval that collapsed genes 30 min on either side of a given time point. For example, the module score for diurnal phase 2 h was calculated using genes from the 1.5 h, 2 h, and 2.5 h phase bins. Only the 0 h module score was calculated using genes from only two time points (0 h and 0.5 h). Dawn is taken as time 0 throughout. It should be noted that the diurnal cycle and the cell cycle are intertwined in Chlamydomonas and that resolving one over the other is not easily achieved. The length of one complete cell cycle is set by light intensity when cells are grown in constant light and may be shorter or longer than 24 h. For ease of comparison across samples, we used diurnal phase as reference.

### Pseudo-time trajectory construction

We constructed pseudo-time trajectories using the R package Monocle (Trapnell et al., 2014). This trajectory reflects the sequence of gene expression changes from one cell to the next and orders the cells based on their similarity. We extracted the raw counts for cells in the selected clusters and normalized them by the estimateSizeFactors and estimateDispersions functions with default parameters. We only retained genes with an average expression over 0.5 and detected in more than 10 cells for further analysis. We determined variable genes by the differentialGeneTest function with a model against the Seurat clusters. We determined the order of cells with the orderCells function and constructed the trajectory with the reduce Dimension function with default parameters. We extracted the pseudo-time for the cells and plotted the pseudo-time in both the UMAP and the linear shaped trajectory. We plotted the diurnal module scores for each cell ordered by pseudo-time in a heatmap. We plotted the cell division module score against the pseudo-time.

### Compilation of gene lists for module score analysis and scRNAseq exploration

We assembled gene lists for the calculation of module score by mining the literature. For the iron deficiency module score, we selected genes expressed >10 Fragments Per Kilobase of transcript per Million mapped reads (FPKM) and showing the stronger induction by Fe limitation from a comparison of RNA-seq data between Chlamydomonas CC-4532 grown in TAP medium + 0.25 µM FeEDTA and TAP medium + 20 µM FeEDTA (Urzica et al., 2012). We extracted the lipid biosynthesis and nitrogen gene lists from (Schmollinger et al., 2014). We ordered normalized expression data from a 48-h time-course in CC-4349 to identify genes that were induced in response to N deficiency (with normalized expression of 0 at 0 h and expression of 1 at 48 h) or repressed by N deficiency (or induced by N sufficiency, with normalized expression of 1 at 0 h and expression close to 0 at 48 h). The lists of lipid biosynthetic genes and ribosome protein genes were according to Supplemental Data Sets 14 and 9 from (Schmollinger et al., 2014), respectively. The photosynthesis gene list include all nucleus-encoded genes from Supplemental Data Set 5 from Strenkert et al. (2019). Cell cycle genes were obtained from Supplemental Data Set 4 of Zones et al. (2015). Genes specific to *mt*^−^ and *mt*^+^ gametes were extracted from Lopez et al. (2015). Finally, we determined the diurnal phase of 10,294 high-confidence rhythmic genes by looking at the overlap between genes deemed to be rhythmic in two separate studies (Zones et al., 2015; Strenkert et al., 2019) and using the diurnal phase values from the 2015 work that had been recalculated for the 2019 study. Gene lists are provided as Supplemental Data Sets 1–10.

### Accession numbers

Sequence data from this article can be found at Phytozome under the following accession numbers: *FEA1* (Cre12.g546550), *FEA2* (Cre12.g546600), *FRE1* (Cre04.g227400), *FOX1* (Cre09.g393150), *FTR1* (Cre03.g192050), *TEF22* (Cre12.g546500), *MSD3* (Cre16.g676150), *CDJ3* (Cre01.g009900), *CGLD27* (Cre05.g237050), *CTP1* (Cre16.g682369), *IRT1* (Cre12.g530400), *IRT2* (Cre12.g530350), *PHC1* (Cre17.g717900), *PHC21* (Cre02.g094450), *VSP1* (Cre11.g467710), *GAS28* (Cre11.g481600), *ACA4* (Cre10.g459200), *MTP1* (Cre03.g145087), and *LCI6* (Cre12.g553350). Other genes used to calculate module scores are listed in Supplemental Data Sets 1–10. scRNA-seq data sets were deposited at Gene Expression Omnibus at NCBI under the accession number GSE157580.

## Supplemental data

The following materials are available in the online version of this article.

**Supplemental Figure 1**. Modules scores for mitochondrial *RPG*s and lipid biosynthetic genes in cells from Experiment 2. (Supports Figure 2).

**Supplemental Figure 2**. The Endogenous diurnal phase of individual cells explains the heterogeneity of batch cell cultures without iron. (Supports Figure 3).

**Supplemental Figure 3**. Pseudo-time construction aligns Fe+ cells along the diurnal cycle. (Supports Figure 4).

**Supplemental Table 1**. Summary of number of cells sequenced, number of genes and UMIs detected.

**Supplemental Table 2**. Summary of the number of genes detected in cells across samples.

**Supplemental Table 3**. Summary of the number of cells expressing a common set of genes across samples.

**Supplemental Data Set 1**. Fe deficiency module score gene list.

**Supplemental Data Set 2**. Nitrogen deficiency module score gene list.

**Supplemental Data Set 3**. Nitrogen sufficiency module score gene list.

**Supplemental Data Set 4**. Chloroplast ribosomal protein gene (*RPG*) module score gene list.

**Supplemental Data Set 5**. Cytosolic ribosomal protein gene (*RPG*) module score gene list.

**Supplemental Data Set 6**. Mitochondrial ribosomal protein gene (*RPG*) module score gene list.

**Supplemental Data Set 7**. Lipid biosynthesis module score gene list.

**Supplemental Data Set 8**. Cell division module score gene list.

**Supplemental Data Set 9**. Photosynthesis module score gene list.

**Supplemental Data Set 10**. Diurnal phase for high-confidence rhythmic genes.

**Supplemental Data Set 11**. *mt*^−^ module score gene list.

**Supplemental Data Set 12**. *mt*^+^ module score gene list.

## Supplementary Material

koab025_Supplementary_Data

## Notes

F.M. and P.A.S. authors contributed equally to this work. P.A.S., M.P. and S.S.M. designed the experiments. P.A.S. grew all Chlamydomonas cultures and collected cells for scRNA-seq. F.M. mapped the reads to the Chlamydomonas genome and analyzed sequencing results. P.A.S. provided gene lists to calculate module scores. P.A.S. and F.M. wrote the manuscript with input from all authors.

The author responsible for distribution of materials integral to the findings presented in this article in accordance with the policy described in the Instructions for Authors (https://academic.oup.com/plcell) is: Matteo Pellegrini (matteop@mcdb.ucla.edu)
