# Supplementary material for: Single-cell RNA sequencing of batch Chlamydomonas cultures reveals heterogeneity in their diurnal cycle phase
Source: Plant Cell. 2021 Feb 2;33(4):1042–57. doi: 10.1093/plcell/koab025 (PMC8226295; doi:10.1093/plcell/koab025)
Supplement: koab025_Supplementary_Data [file koab025_supplementary_data.zip › tpc.00762.2020-s01.pdf]

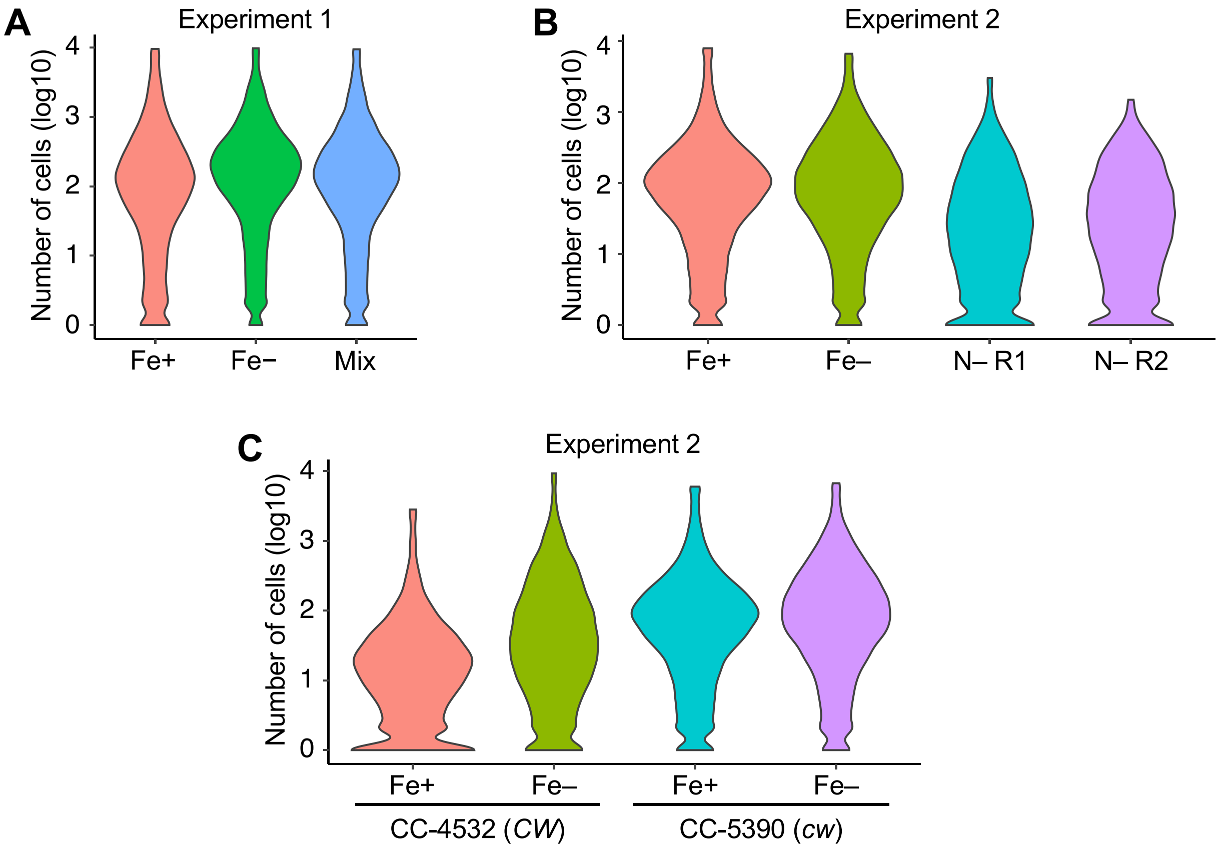

Supplemental Figure 1. Distribution of the number of cells expressing a given gene. (Supports Figures 1, 2, and 3).

For each experiment and sample, we determined the number of cells in which a gene is deemed expressed. We show the distribution as a violin plot. At the lower end of the violin plots, some genes are seldom detected. The bulk of genes is detected in ~100-500 cells from the same samples. In the upper end of the distributions, some genes are expressed and detected across most cells.

(A) Distribution of the number of genes expressed across cells from experiment 1.

(B) Distribution of the number of genes expressed across cells from experiment 2, for iron (Fe) and nitrogen (N) deficiency samples.

(C) Distribution of the number of genes expressed across cells from experiment 2, comparing CC-4532 (with cell wall, *CW*) and CC-5390 (withouth cell wall, *cw*).

The numbers are also provided in Supplemental Table 2.

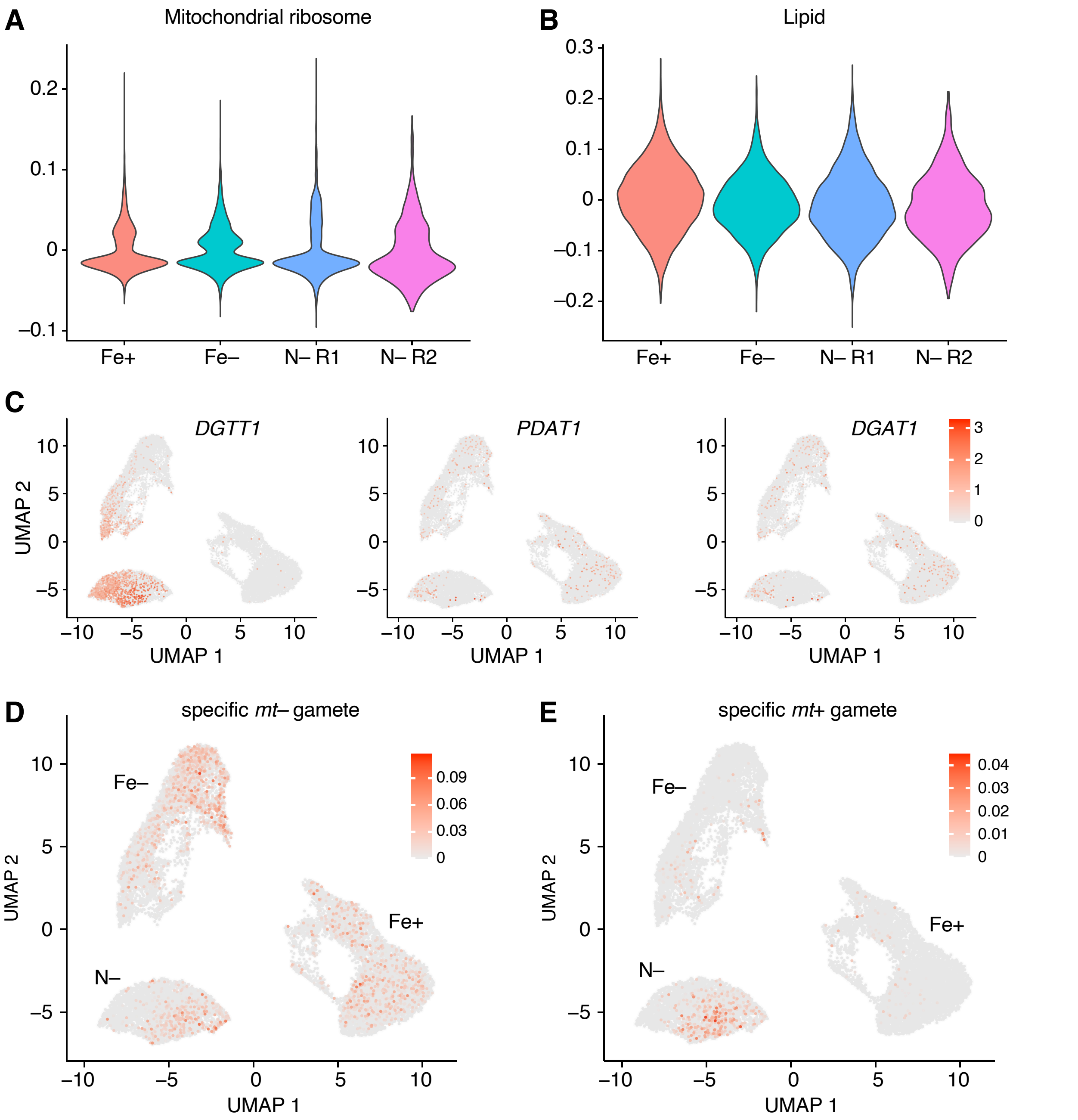

Supplemental Figure 2. Modules scores for mitochondrial *RPG*s, lipid biosynthetic genes and the gametic program in cells from experiment 2. (Supports Figure 2).
(A) Mitochondrial *RPG* module score for each sample.
(B) Lipid module score for each sample, using a gene list compiled by Schmollinger et al. (Schmollinger et al., 2014).
(C) UMAP plots for the triacylglyceride (TAG) biosynthesis-related genes *DGGT1* (Cre12.g557750), *DPAT1* (Cre02.g106400) and *DGAT1* (Cre01.g045903).
(D) Module score for genes specifically expressed in *mt*– gametes, from Lopez et al. (Lopez et al., 2015).
(E) Module score for genes specifically expressed in *mt*+ gametes, from Lopez et al. (Lopez et al., 2015).

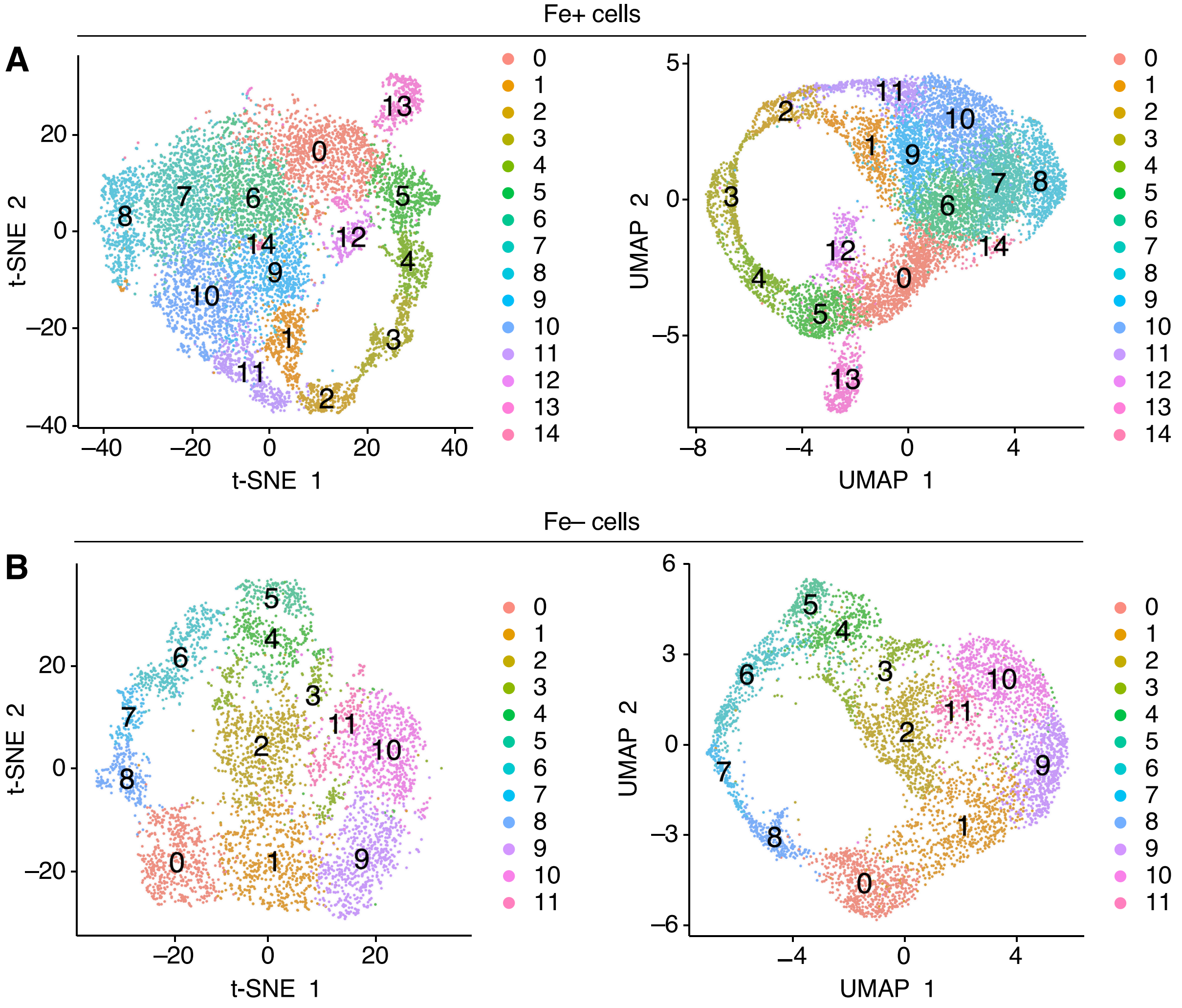

Supplemental Figure 3. t-SNE and UMAP plot of cultures grown in iron-sufficient and -deficient conditions. (Supports Figure 3).

(A, B) t-SNE (**A**) and UMAP (**B**) plot of 9,517 sequenced cells from experiment 1 that were grown in Fe+ condition for 23 h. The cells were separated into clusters by Seurat (Stuart et al., 2019) and are indicated by the color gradient, with the color key on the right side of the plot.

(C, D) t-SNE (**C**) and UMAP (**D**) plot of 9,748 sequenced cells from experiment 1 that were grown in Fe– condition for 23 h. The cells were separated into clusters by Seurat (Stuart et al., 2019) and are indicated by the color gradient, with the color key on the right side of the plot.

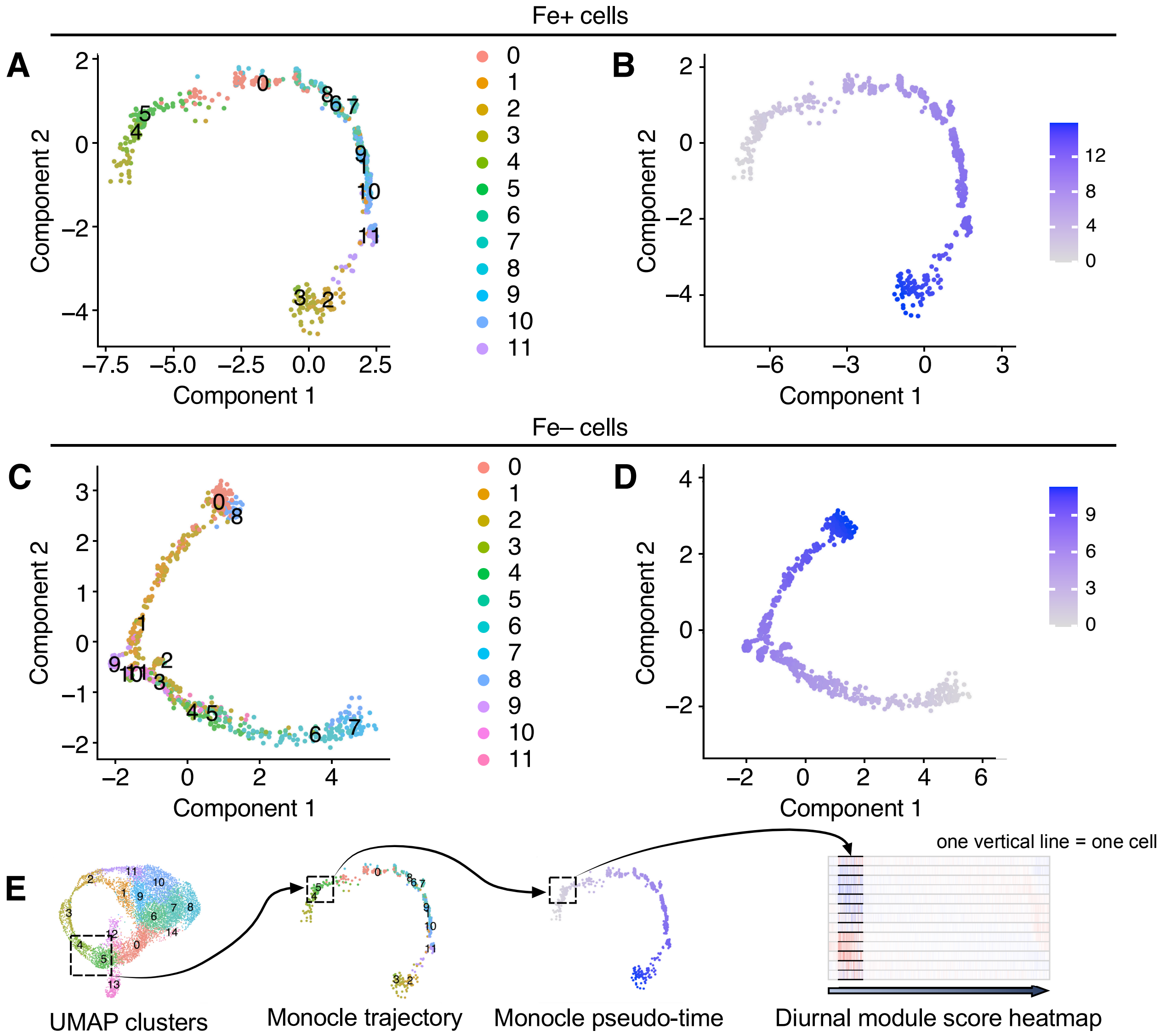

Supplemental Figure 4. Pseudo-time construction aligns cells along the diurnal cycle. (Supports Figure 4).

(A) Trajectory plot of Fe+ cells from experiment 1, colored according to their constituent clusters. Replotted from Figure 3 for reference.

(B) Trajectory plot of Fe+ cells from experiment 1, colored according to their pseudo-time. The plots in (**A**) and (**B**) are identical but colored based on the clusters they belong to (**A**) or according to their pseudo-time (**B**).

(C) Trajectory plot of Fe– cells from experiment 1, colored according to their constituent clusters. Replotted from Figure 3 for reference.

(D) Trajectory plot of Fe+ cells from experiment 1, colored according to their pseudo-time. The plots in (**C**) and (**D**) are identical but colored based on the clusters they belong to (**C**) or according to their pseudo-time (**D**).

(E) Principle of cell alignment along their pseudo-time based on Monocle trajectory results.

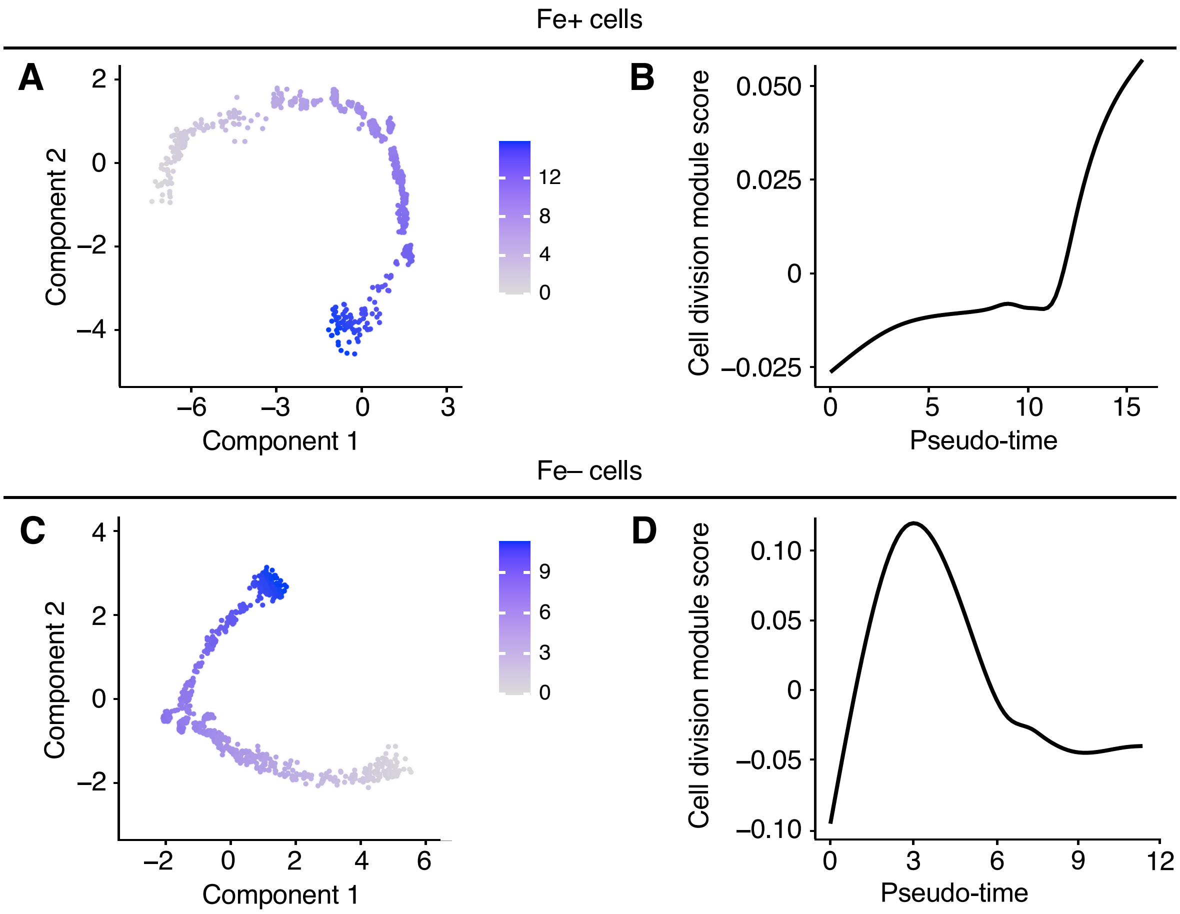

Supplemental Figure 5. Cell division score in Fe+ and Fe– cells from experiment 1 as a function of pseudo-time.

(A, C) Pseudo-time trajectory of Fe+ (**A**) and Fe– (**B**) cells from experiment 1, colored according to their pseudo-time. Replotted from Supplemental Figure 4 as reference.

(B, D) Cell division module score, plotted as a function of pseudo-time, for Fe+ (**C**) and Fe– (**D**) cells from experiment 1.

Supplemental Table 1. Summary of number of cells sequenced, number of genes and UMIs detected.

Experiment	Sample	Strain	Number cells	UMIs per cell	Genes per cell
1	Fe+	CC-5390 (*cw*)	9,517	3,009	827
1	Fe–	CC-5390 (*cw*)	9,748	3,716	828
1	Mix	CC-5390 (*cw*)	9,425	3,297	815
2	Fe+	CC-5390 (*cw*)	7,960	4,044	630
2	Fe–	CC-5390 (*cw*)	6,656	5,380	747
2	Fe+	CC-4532 (*CW*)	2,814	3,961	409
2	Fe–	CC-4532 (*CW*)	9,289	2,757	414
2	N– R1	CC-5390 (*cw*)	3,028	2,034	578
2	N– R2	CC-5390 (*cw*)	1,496	3,924	1,035

Total	59,933

Supplemental Table 2. Summary of the number of genes detected in cells across samples.

	Experiment 1				Experiment 2						
Number of genes	Fe+	Fe–	Mix	Merge 1*	Fe+	Fe–	N– R1	N– R2	Merge 2*	CW Fe+	CW Fe–
Min	385	201	312	201	240	176	178	174	174	134	122
Max	3,232	5,870	2,729	5,870	2,966	3,377	3,828	3,783	3,828	1,881	3,998
Mean	827	828	815	823	630	747	578	1038	694	410	414
Median	767	773	754	763	550	678	400	796	597	380	313
Total number of genes	16,080	16,638	16,380	16,982	16,481	16,505	15,096	15,206	17,039	14,643	15,768

*Merge1 contains the Fe+, Fe– and Mix sample from experiment 1.
*Merge2 contains the Fe+, Fe–, N– R1 and N– R2 sample from experiment 2.

Supplemental Table 3. Summary of the number of cells expressing a common set of genes across samples.

	Experiment 1				Experiment 2						
Number of cells	Fe+	Fe–	Mix	Merge 1*	Fe+	Fe–	N– R1	N– R2	Merge 2*	CW Fe+	CW Fe–
Min	1	1	1	1	1	1	1	1	1	1	1
Max	9,517	9,748	9,425	28,690	7,960	6,656	3,028	1,496	19,140	2,814	9,289
Mean	463	475	453	1,391	294	292	103	91	780	68	225
Median	100	150	119	379	76	77	20	24	223	12	33
Total number of cells	9,517	9,748	9,425	28,690	7,960	6,656	3,028	1,496	19,140	2,814	9,289

*Merge1 contains the Fe+, Fe– and Mix sample from experiment 1.
*Merge2 contains the Fe+, Fe–, N– R1 and N– R2 sample from experiment 2.
