# Supplementary material for: Single-cell RNA sequencing of batch Chlamydomonas cultures reveals heterogeneity in their diurnal cycle phase
Source: Plant Cell. 2021 Feb 2;33(4):1042–57. doi: 10.1093/plcell/koab025 (PMC8226295; doi:10.1093/plcell/koab025)
Supplement: koab025_Supplementary_Data [file koab025_supplementary_data.zip › tpc.00762.2020-s03.pdf]

Single-Cell RNA Sequencing of Batch Chlamydomonas Cultures Reveals Heterogeneity in their Diurnal Cycle Phase

Corresponding author: Patrice Salomé psalome@aspb.org

Review timeline:

**TPC2020-RA-00762**	Submission received:	September 16, 2020
	1st Decision:	December 2, 2020 *revision requested*
**TPC2020-RA-00762R1**	1st Revision received:	January 1, 2021
	2nd Decision:	January 3, 2021 *acceptance pending, sent to science editor*
	Final acceptance:	January 13, 2021

REPORT: (The report shows the major requests for revision and author responses. Minor comments for revision and miscellaneous correspondence are not included. The original format may not be reflected in this compilation, but the reviewer comments and author responses are not edited, except to correct minor typographical or spelling errors that could be a source of ambiguity.)

TPC2020-RA-00762 1st Editorial decision – *revision requested* December 2, 2020

We ask you to pay attention to the following points in preparing your revision:

Reviewer 1 recommended declining the manuscript and merging the results with the companion paper on co-expression networks in Chlamy. However, the other two reviewers viewed the manuscript more favorably. Reviewer 2 noted that it is mainly a "methods" paper, but quite valuable for that, as one of the early papers to report single-cell RNA-seq in Chlamy cultures. Reviewer 3 judged that the work will have a significant impact on researchers in the field of chronobiology, and will provide an important basis for future studies scRNA-seq in Chlamy. The editors find that the two manuscripts do stand on their own, and should be published as two papers. This scRNA-seq manuscript is perhaps the weaker of the two; the extent to which you can address Reviewer 1 and 2 comments in a revision can strengthen this manuscript considerably.

Please address the major comments of Review 1 (specifically #2, 3, 4), and comments of reviewer 2 (in general, provide a better exploration of the technical aspects; specifically, address the 6 numbered comments). These reviewer comments need to be addressed in some manner in the manuscript (i.e. not only in a response to reviewer comments document).

-- Reviewer comments:

<u>Reviewer #1:</u>

This manuscript by Patrice Salomé reports single cell RNA-seq (scRNA-seq) in cultures of Chlamydomonas that have been deprived of iron (Fe) or nitrogen (N). Previously, the RNA-seq signature for these nutrient stresses have been reported using bulk RNA-seq (and the data sets and their analysis are publicly available), resulting in a known "signature" of these stresses in Chlamydomonas cells. Using scRNA-seq, the authors recapitulate this finding for both Fe and N deprivation in large cell populations. During analysis of their data they found that their cultures grown in the presence of acetate under 24h of light clustered in a "cycle". Subsequent data analysis demonstrated that they could recapitulate cell populations in various stages of their cell cycles, and a rhythmic pattern of gene expression could be reconstructed. The primary conclusion is that batch grown cells, even when nutrient stressed, have an significant cell cycle signature of gene expression in addition to the nutrient deprivation response.

Overall, the experiments appear to have been carefully performed and analyzed. Unfortunately, I think that while the work itself is high quality, the finding does not significantly advance our understanding of plant or agal biology. The techniques are being newly utilized in Chlamydomonas, but are not themselves novel.

Below are the points for the authors to consider to improve the manuscript.

Major revisions:

1) The fundamental finding in this paper it a well-known phenomenon for those that study the Chlamydomonas cell cycle. That cells in batch culture under 24h of light with acetate have populations of cells in various stages of the cell cycle. It is this asynchronous aspect to cell populations that cell cycle researchers try and break with various techniques, including light cycle control, N starvation followed by photo autotrophic media, and possibly drug treatments. In fact, there have been reports at various venues that heterogeneity in Chlamydomonas cultures is common and may affect certain experiment types, including transformation and some nutrient experiments. It is nice to see that this finding is recapitulated in these data sets and may indeed be a warning to other researchers about the possible need for culture synchrony rather than asynchronous batch cultures. However, while it is reassuring to see this recapitulated by scRNA-seq, this finding alone is not new to the field.

2) Nitrogen deficiency should have induced the gametic program, and I don't see this finding. The gametic program should have an obvious and strong signature in this experiment. I have a strong secondary concern, that the cells in the experiment are described as "chlorotic". This suggests something may be fundamentally incorrect about how the experiment was done (too high of light?, pre-starved for nutrients? other?).

3) The statistical analysis of the experimental results appears to be absent. There are numerous ways that the statistical power of scRNA-seq experiments can be addressed. In particular, Supplemental Table 1 is not very informative. On its face, it appears that the scRNA-seq coverage is low for the conclusions that were derived. There are at least two ways to determine statistical power (e.g. Zhang et al 2020 DOI: 10.1038/s41467-020-14482-y) if not others. This is a major issue with low coverage scRNA-seq data and dimensionality reduction. Just because the dimensional reduction derives an answer, does not necessarily mean that the results are significant.

4) The RNA extractability results appear to be important, but supplemental in nature and could be discussed in only the results or strictly in the supplement. Given the protein composition of the Chlamydomonas cell wall, and significant lack of cell wall in *cw* strains does not make this much of a surprising result given how easy it is to make bulk-RNA-seq libraries.

<u>Reviewer #2:</u>

This paper examines whether single-cell RNA sequencing methods can be applied to Chlamydomonas. This is something worth knowing, and the answer looks positive (though with many of the same caveats that apply to scRNA in other systems). Some biological findings are presented but mainly this is a methodological paper.

1) In the first experiment presented, information was obtained about an average of 823 genes each in 30,000 cells. It is not stated the average number of genes for which information was obtained in each cell, nor the histogram of how many genes were identified in more than one cell. Consider some extremes (obviously neither is correct!): all cells had information about one gene, but almost no other genes were detected in more than a few cells (something like this could happen if one or a few genes were very highly expressed and the rest quite low, which in fact is a true feature of transcriptomes in all organisms); opposite extreme: all 823 genes gave read counts in all 30,000 cells. We can't tell which, and it does matter rather a lot.

Anyway, no matter how you cut it, the vast majority of genes in the organism are unsampled, in a given cell and/or in the population overall. And in most scenarios, the simple idea that 'gene A and gene B are co-expressed' might be a challenging statement to make with confidence if it should be the case that in no cell are there reads detected for both A and B. (Such claims nevertheless ARE made in some scRNA studies based on the t-SNE approach; it may be correct but it seems risky to me).

On the other hand, perhaps I totally misunderstand because the statement is then made (line 136) 'Since the scRNA-seq dataset consisted of expression information from 16,982 genes across about 30,000 cells,...'. Since this is about the total number of genes in the genome, does this mean that in aggregate all genes are covered in some cell or other, or does it mean that there COULD have been information about this many genes even if some were never detected at all?

I realize these problems with coverage are generic to scRNA but that doesn't make them go away. I'm not saying there's a problem here, just that the presentation doesn't allow me to evaluate.

2) The UMAP method is used to reduce dimensionality, obviously a critical step but explained not at all here. Since the method is new (~2019), it is unreasonable to expect the reader to (1) know what it is; (2) unconditionally accept its applicability. It is, I gather, a stochastic neighbor-joining method, as is the t-SNE method. t-SNE has up to recent past been a standard way to analyze scRNA datasets; I accept that t-SNE is useful but I think it is also perilous (the 'S' stands for stochastic, and depending on details you can get very different answers with different runs. There can be in addition very strong dependence on parameters). Whether UMAP has the same issues, and/or what other issues it might have, should be discussed up front. Both t-SNE and UMAP are described (in various semi-comprehensible websites I looked at) as 'visualization' tools, not analytical tools (unlike PCA); it is apparently clear that the relative locations of t-SNE clusters is essentially uninformative and it is still under discussion what is the status of UMAP in this regard. This matters, for example in the discussion of the circle of clusters. If relative position on the UMAP1 x UMAP2 plot is uninformative, then so is the circle. Please understand, I'm not saying this is the case, I really don't know about the method; but since it's a critical tool the burden is on the authors to justify its use.

Since essentially every figure includes UMAP coordinates, from a certain point of view I am simply unable to evaluate almost anything in the paper. However, I'll do what I can.

3) It is shown that scRNA can separate Fe+ from Fe-deprived gene expression patterns. This works with UMAP; Fig. 1C and 1D seem to show that it works just about as well with a simple indexing procedure, but I believe the implication is that UMAP works without any prior information, unlike the indexing approach. In any case, it does show that scRNA can at least distinguish broad states of the cell; unclear to me how many differentially expressed genes, and what degree of differential regulation, is required. This the authors could evaluate computationally; e.g. if the datasets are truncated by removing X% of Fe-regulated genes, how high can X be before Fe+ and Fe- cells can't be distinguished? This would be useful for people trying to evaluate if the UMAP method might be useful in a situation with perhaps less powerful (and previously unknown) gene expression signatures.

4) Clustering of the Fe (randomly cycling in continuous light) showed a subset of clusters that formed a ring; intriguingly progression around the ring was associated with an increasing diurnal index from previous work. This is interesting, and discussed sensibly. It does bother me some that while this is true of 5 of the clusters, 8 of the clusters are NOT on the ring, and their diurnal score is not presented. These represent at least half the total cells, I would guess - so are these cells NOT at some random diurnal position, but in some off-state? What's going on? This is not discussed that I can see. There does appear to be relevant information in Fig. 3C, but this is not explained in a way that helps me much.

5) I'm sorry to say that I really don't understand the 'pseudo-time' assignment. It seems to me redundant with the diurnal assignment of clusters already carried out. Somewhat as with UMAP, it appears that you can only understand this analysis by knowing something about a program called Monocle. I never heard of it, and unless everyone else in the TPC readership is like 'Of course, Monocle, what else would you use?', I think the authors of the present MS ought to explain what this program does and why it is appropriate.

6) The paper shows that the methodology works with cells with or without cell walls, which is technically a helpful thing, and further, that cell-wall-less strains have their own (UMAP!) gene signature.

Overall, the authors have done a useful thing by validating scRNA in Chlamydomonas, and I am generally convinced by their controls. The diurnal/random phase in continuous light idea is interesting but seemingly incomplete given that 8/13 clusters don't fit the picture (or else I just misunderstand). In general, for a paper whose primary purpose is technical evaluation of a method, technical aspects are under-explored - prime examples are the degree and consequences of undersampling, and the use of powerful computer programs that do things to the data that the reader (at least this reader) can't figure out to produce visually appealing but less-than-fully-informative images.

Reviewer #3:

The manuscript described the scRNA-seq method in the model green alga *Chlamydomonas reinhardtii*. The authors performed scRNA-seq analysis in Chlamydomonas under iron- and nitrogen-deficient conditions and obtained results that were in good agreement with previous findings, demonstrating that the method works in Chlamydomonas. They also found that heterogeneity existed among individual cells under iron-replete and iron-deficient conditions and showed that this can be well explained by diurnal variation in gene expression. Finally, it is shown that the method can be applied to cells with and without cell walls.

The applicability of the scRNA-seq method to Chlamydomonas is well supported by the results obtained here. It is also interesting that the asynchronous cultures of Chlamydomonas contain cells of various diurnal phases. In general, conventional analysis using RNA from the entire population of asynchronous cultures (Northern blot, RT-qPCR) result in the loss of circadian rhythmicity during long-term cultivation without entrainment stimuli. This phenomenon is explained by the asynchrony among individual cells, as has been demonstrated by the analysis of specific reporter gene rhythms in other model systems. It has been believed that the situation is the same in Chlamydomonas. This study demonstrated this point clearly by global gene expression, rather than by a limited number of reporter genes. This is expected to have a significant impact on researchers in the field of chronobiology. Furthermore, the findings of this study will provide an important basis for future studies using scRNA-seq method in Chlamydomonas.

Major points

None

Minor points

The heterogeneity appears smaller under nitrogen-depleted conditions than under other conditions (Fe+, Fe-). It is also interesting that there is no cyclic distribution under nitrogen-depleted conditions. Does the diurnal rhythm disappear under nitrogen-deficient conditions? The authors might want to mention this point.

line 249: This sentence seems to be a description of S Fig 1B, not S Fig 2B.

TPC2020-RA-00762R1 1st Revision received January 1, 2021

Reviewer comments and **author responses**:

We appreciate the detailed and constructive comments and suggestions from the editors and the reviewers. The manuscript has been carefully revised, and the point-by-point answers to the comments and suggestions are listed below.

In short, we provide explanations of the t-SNE and UMAP methods, as well as their possible limitations. We also looked at the gametic program in N-limited cells: it appears to be on, although the time point used here (24 h) is unlikely to have captured earlier events, as previous work on the topic considers ~8 h post transfer to N– conditions late in the process. The decrease of chlorophyll content has been published in multiple studies. We have also gone back to the clustering analysis and realized that all clusters were strongly rhythmic, thus extending our initial conclusions.

We have modified Figure 1 to include the t-SNE plot before the UMAP plot or Fe+ and Fe– cells. We have replaced Figures 3 and 4 with new versions to reflect the extended diurnal time analysis. We have added Supplemental Figure 1 and Supplemental Tables 2 and 3 to provide the number and distribution of genes sequences and the number of cells in which they were detected.

Reviewer #1:

Point 1. The fundamental finding in this paper is a well-known phenomenon for those that study the Chlamydomonas cell cycle. That cells in batch culture under 24h of light with acetate have populations of cells in various stages of the cell cycle. It is this asynchronous aspect to cell populations that cell cycle researchers try and break with various techniques, including light cycle control, N starvation followed by photo autotrophic media, and possibly drug treatments. In fact, there have been reports at various venues that heterogeneity in Chlamydomonas cultures is

common and may affect certain experiment types, including transformation and some nutrient experiments. It is nice to see that this finding is recapitulated in these data sets and may indeed be a warning to other researchers about the possible need for culture synchrony rather than asynchronous batch cultures. However, while it is reassuring to see this recapitulated by scRNA-seq, this finding alone is not new to the field.

RESPONSE: We have two points to offer in response to the reviewer. First, it may well be reported that batch cultures are variable, but we do not believe that this is strongly documented with experiments; rather it represents anecdotal assumption in the community.

It is one thing to hypothesize variability in batch cultures or to suggest it based on visual inspection of cultures where cells are differentiated by size or by the presence of some fraction of recently divided cells; it is another to document the transcriptome of thousands of individual cells to substantiate the claim. Therefore, we believe that our results will be important to the Chlamydomonas community, and beyond, for future experimental design. We have slightly modified the abstract to reflect this point (Lines 50-51).

Second, we point out that the data set suggests that individual cells are in fact not entirely heterogenous, and that many cells share the same diurnal phase despite having been grown in constant light and dividing more frequently than once a day as in typical diurnal experiments. With the obvious caveat that a cell cycle-driven rhythmic component cannot be distinguished from a truly diurnal or circadian-based rhythm in Chlamydomonas, this observation suggests that a large component of the diurnal rhythm is likely related to progression through the cell cycle, whose timing is retained for a significant number of cells after inoculation into fresh medium, or that there is a social signaling mechanism. This observation goes well with the extended analysis of the clusters and pseudo-time trajectories, in response also to reviewer #2 (Lines 357-370).

Point 2. Nitrogen deficiency should have induced the gametic program, and I don't see this finding. The gametic program should have an obvious and strong signature in this experiment.

RESPONSE: We have included citations to the Lopez et al., 2015 Plant Physiology and Abe et al., 2004, Current Genetics papers where expression of genes in the gametic program are identified and defined. Abe et al. noted that gamete-specific genes are strongly up-regulated very shortly after cells experience N-deficiency and the response is transient so that most genes in the gametic background are not substantially increased in expression after 24 h, which is a very late time point. We do note mild increases in the abundance of a fraction of transcripts, and this is noted in the discussion. Our gene lists consist of 35 *mt*– genes and 145 *mt*+ genes from Lopez et al., as well as 18 *NSG* genes from Abe et al. (only one *NSG* gene also belongs to the *mt*+ list, so we treated each gene list separately). Of those, 14/35 (*mt*– genes) and 33/145 (mt+ genes) were induced by low N, reaching their peak between 8-12 h after transfer to N deficiency conditions.

We have nevertheless added an analysis of gamete-specific genes in Supplemental Figure 1, using a gene list from Lopez et al., 2015, Plant Physiology, http://www.plantphysiol.org/content/169/4/2730). We do observe a signal in N deficient cells for genes normally associated with the development of *mt*+ gametes, consistent with the genotype of the strain used here (CC-5390, *mt*+). However, we also note that few gametic genes are induced under long-term low N conditions, based on Schmollinger et al., 2014. As mentioned above, genes suspected to be involved in the gametic program are induced within 2-5 h of N deficiency, with genes induced 5-8 h into the deficiency being considered "late expressed", as for example in Abe et al., 2004, Current Genetics, https://pubmed.ncbi.nlm.nih.gov/15459796/).

Since we maintained our cultures in N deficiency conditions for 24 h, we hypothesize that the gametic program is largely no longer induced and that cells have differentiated, although we did not test mating competence in these cells at the time of the experiment. This point has now been added to the results (Lines 296-328).

I have a strong secondary concern, that the cells in the experiment are described as "chlorotic". This suggests something may be fundamentally incorrect about how the experiment was done (too high of light?, pre-starved for nutrients? other?).

RESPONSE: Chlorosis is an adjective describing the color of the cells (namely light green). Any nutrient deficiency will result in chlorosis, especially N-deficiency. Indeed, this phenotype is classically referred to as N-deficiency chlorosis. The 1989 paper by Plumley and Schmidt (PNAS, https://www.pnas.org/content/pnas/86/8/2678.full.pdf) says in the first sentence of the abstract, "Nitrogen-limited Chlamydomonas is chlorotic". We have also previously described the down-regulation of chlorophyll-proteins at the level of the proteome and transcriptome in batch culture

in previous work as well (Schmollinger et al., 2014; http://www.plantcell.org/content/plantcell/26/4/1410.full.pdf), in which we observed a 65% decrease in chlorophyll levels per cell after 24 h in N-deficient conditions. We have included a few sentences in the introduction to describe the prior work by Plumley and Schmidt as well as by Schmollinger et al. where chlorosis is discussed in the context of chlorophyll-protein abundance. This point is now made clear in results (Lines 300-311).

Point 3. The statistical analysis of the experimental results appears to be absent. There are numerous ways that the statistical power of scRNA-seq experiments can be addressed. In particular, Supplemental Table 1 is not very informative. On its face, it appears that the scRNA-seq coverage is low for the conclusions that were derived. There are at least two ways to determine statistical power (e.g. Zhang et al 2020 DOI: 10.1038/s41467-020-14482-y) if not others. This is a major issue with low coverage scRNA-seq data and dimensionality reduction. Just because the dimensional reduction derives an answer, does not necessarily mean that the results are significant.

RESPONSE: In scRNA-seq analysis, the statistical power may be low to determine whether one gene is differentially expressed between two groups, either due to low expression of the gene or the technical dropout issue. In our study, the low expression and the dropout issue were overcome by computing an aggregated module score across a list of genes. Since we are comparing these aggregate scores between thousands of cells, the statistical power is significant.

We performed a Wilcoxon Rank Sum Test on the iron deficiency module scores comparing the Fe+ and Fe– cells, and the p value was smaller than the machine epsilon 2.2e-16, indicating a very significant difference.

We added the statistical test and p value to the legend for Figure 1E.

Thank you for the opportunity to explain this point.

Point 4. The RNA extractability results appear to be important, but supplemental in nature and could be discussed in only the results or strictly in the supplement. Given the protein composition of the Chlamydomonas cell wall, and significant lack of cell wall in *cw* strains does not make this much of a surprising result given how easy it is to make bulk-RNA-seq libraries.

RESPONSE: While it is true that RNA has been extracted from many Chlamydomonas strains for bulk RNA-seq analysis, the RNA extraction protocol usually employs ionic detergents and proteases. Our standard laboratory protocol for bulk extraction from cultures works well for strains without a cell wall (like CC-5390), as well as with strains with a moderate (CC-4532) and strong (CC-1690) cell wall, but requires the use of 2% SDS to lyse the cells as rapidly as possible to limit RNA degradation by ribonucleases. We now make the distinction between the detergent-containing protocol for bulk cultures vs. the 10X protocol in the results.

In addition, we note that many phycologists are attempting scRNA-seq with walled algal cells and we believe the fact that one can extract information even in walled algal cells from the standard 10X pipeline may be informative to that audience. At the same time, it is useful to understand the technical differences between walled and wall-reduced (*cw*) strains, since so many researchers use genotypes in one or the other background.

It is possible to remove walls with enzymes (for Chlamydomonas many use autolysin), but of course then there is a delay in capturing the transcript pool, which may have changed in the meantime. This is standard for looking at cell type specific expression in a developmental context in plants, where the timing required for enzyme treatment may not have a big impact, but to capture early responses to nutrient or other signals (e.g. light quality or quantity) in a situation where mRNA half-lives are on the order of minutes, it is useful to have the option of immediate sampling of cells in a culture. There is also the option of capturing mRNA profiles by freezing material for subsequent treatment, and this would require more methodological development, which is in progress.

These points have been emphasized in the results (Lines 421-426) and discussion (Lines 541-544 and 557-560).

Reviewer #2:

This paper examines whether single-cell RNA sequencing methods can be applied to Chlamydomonas. This is something worth knowing, and the answer looks positive (though with many of the same caveats that apply to scRNA in other systems). Some biological findings are presented but mainly this is a methodological paper.

Point 1. In the first experiment presented, information was obtained about an average of 823 genes each in 30,000 cells. It is not stated the average number of genes for which information was obtained in each cell, nor the histogram of how many genes were identified in more than one cell. Consider some extremes (obviously neither is correct!): all cells had information about one gene, but almost no other genes were detected in more than a few cells (something like this could happen if one or a few genes were very highly expressed and the rest quite low, which in fact is a true feature of transcriptomes in all organisms); opposite extreme: all 823 genes gave read counts in all 30,000 cells. We can't tell which, and it does matter rather a lot.

Anyway, no matter how you cut it, the vast majority of genes in the organism are unsampled, in a given cell and/or in the population overall. And in most scenarios, the simple idea that 'gene A and gene B are co-expressed' might be a challenging statement to make with confidence if it should be the case that in no cell are there reads detected for both A and B. (Such claims nevertheless ARE made in some scRNA studies based on the t-SNE approach; it may be correct but it seems risky to me).

On the other hand, perhaps I totally misunderstand because the statement is then made (line 136) 'Since the scRNA-seq dataset consisted of expression information from 16,982 genes across about 30,000 cells,...'. Since this is about the total number of genes in the genome, does this mean that in aggregate all genes are covered in some cell or other, or does it mean that there COULD have been information about this many genes even if some were never detected at all?

I realize these problems with coverage are generic to scRNA but that doesn't make them go away. I'm not saying there's a problem here, just that the presentation doesn't allow me to evaluate.

RESPONSE: We realize it is challenging to present a complete summary of the number of genes detected in each cell across each sample. The second plot in Figure 1A shows the distribution of how many genes were detected in individual cells, and this number ranged from 500 to 2,000. We found that on average each gene was detected in 463, 475 and 452 cells in the Fe+, Fe– and Mix sample, respectively, and in 1,391 cells across all three samples. Most of the genes were detected in fewer than 2,500 cells in each library, with a small fraction of genes detected in almost every cell in each library.

The statement 'Since the scRNA-seq dataset consisted of expression information from 16,982 genes across about 30,000 cells...' means that 16,982 genes were detected in at least one cell. We fully understand the urge to try and quantify in simple terms the high complexity of scRNA-seq datasets, and now provide Supplemental Tables 2 and 3 with more numbers, as well as a new Supplemental Figure 1 that shows the distribution of the number of cells in which a given gene was deemed expressed in all our samples. Please note that experiment #2 used a different sequencing platform with distinct attained coverage, and should therefore not be directly compared to experiment #1 in terms of absolute numbers.

Point 2. The UMAP method is used to reduce dimensionality, obviously a critical step but explained not at all here. Since the method is new (~2019), it is unreasonable to expect the reader to (1) know what it is; (2) unconditionally accept its applicability. It is, I gather, a stochastic neighbor-joining method, as is the t-SNE method. t-SNE has up to recent past been a standard way to analyze scRNA datasets; I accept that t-SNE is useful but I think it is also perilous (the 'S' stands for stochastic, and depending on details you can get very different answers with different runs. There can be in addition very strong dependence on parameters). Whether UMAP has the same issues, and/or what other issues it might have, should be discussed up front. Both t-SNE and UMAP are described (in various semi-comprehensible websites I looked at) as 'visualization' tools, not analytical tools (unlike PCA); it is apparently clear that the relative locations of t-SNE clusters is essentially uninformative and it is still under discussion what is the status of UMAP in this regard. This matters, for example in the discussion of the circle of clusters. If relative position on the UMAP1 x UMAP2 plot is uninformative, then so is the circle. Please understand, I'm not saying this is the case, I really don't know about the method; but since it's a critical tool the burden is on the authors to justify its use.

Since essentially every figure includes UMAP coordinates, from a certain point of view I am simply unable to evaluate almost anything in the paper. However, I'll do what I can.

RESPONSE: We apologize for the lack of clear explanation of the UMAP method. We note that a cursory look at four recent scRNA-seq publications in Arabidopsis showed that they largely gloss over t-SNE and Monocle analysis. We

see this gap as an opportunity to provide a modicum of explanations of these analysis methods. We have added a paragraph in the introduction to this effect. We think placing this paragraph there rather than in the results section will not break the flow of the results, and perhaps serve the journal readership as more scRNA-Seq work gets published (Lines 90-118).

UMAP and t-SNE are two of the most widely used dimension reduction methods for scRNA-seq analysis. As noted by this reviewer, UMAP is more recent but was used previously by Jean-Baptiste et al., 2019, Plant Cell www.plantcell.org/cgi/doi/10.1105/tpc.18.00785). In the Becht et al 2019 paper that introduced UMAP to this field, the authors demonstrated that UMAP provides higher reproducibility and a more meaningful organization of cell clusters among all the methods they compared, including t-SNE. UMAP also preserved the global structure of the data and the continuity of cell subsets better than t-SNE in this initial report.

Since then, a more recent article (Heiser and Lau, 2020, Cell Reports) compared the performance of 11 widely used dimensionality reduction methods in terms of preservation of structure and relationships using real-world and simulated datasets of two natures: discrete and continuous. UMAP generally performed better with continuous data (to which our cultures are equivalent when considering them one at a time) than t-SNE. However, the relative position and orientation of clusters between samples was not always representative of the underlying data. Accordingly, we used UMAP to determine the clustering behavior of our samples, and then zoomed in on a single group of cells from a single sample, under which conditions UMAP performs very well.

Thus, we believe that the arrangement of cells along a circle, as determined by UMAP on Fe+ and Fe– cells likely reflects an underlying gradient in the cell population, which we believe comes from the endogenous diurnal phase of each cluster, or from their phase along the cell cycle. In support of this hypothesis, we obtained a similar pattern when running tSNE.

We now show the t-SNE plots alongside the UMAP plots in Figure 1 and Supplemental Figure 3, and use them to illustrate the methods in the Results section (Lines 168-182).

Point 3. It is shown that scRNA-seq can separate Fe+ from Fe-deprived gene expression patterns. This works with UMAP; Fig. 1C and 1D seem to show that it works just about as well with a simple indexing procedure, but I believe the implication is that UMAP works without any prior information, unlike the indexing approach. In any case, it does show that scRNA-seq can at least distinguish broad states of the cell; unclear to me how many differentially expressed genes, and what degree of differential regulation, is required. This the authors could evaluate computationally; e.g. if the datasets are truncated by removing X% of Fe-regulated genes, how high can X be before Fe+ and Fe- cells can't be distinguished? This would be useful for people trying to evaluate if the UMAP method might be useful in a situation with perhaps less powerful (and previously unknown) gene expression signatures.

RESPONSE: The embedding of the cells on the UMAP was determined by the global expression pattern. The different locations of the cells were mainly determined by the most differentially expressed genes, removing these genes from the dataset would bring the cells in different states closer to each other on the UMAP. However, it is hard to determine how many genes should be removed to bring the cells together completely. Among the differentially expressed genes, some are significant in the statistical test, and some are not. If we remove the significantly differentially expressed genes from the datasets, the other genes with less difference will still separate the cells on the UMAP.

We performed differential expression analysis between the Fe+ and Fe– cells using the Wilcoxon Rank Sum Test, and controlled the false discovery rate using the Benjamini–Hochberg procedure. Using adjusted p value < 0.05 as a cutoff, we identified 1,589 genes as significantly expressed between the Fe+ and Fe– cells. Interestingly, 69 out of 100 iron deficiency induced genes used in the module score calculation were found in the differentially expressed gene list (Lines 201-204).

Point 4. Clustering of the Fe (randomly cycling in continuous light) showed a subset of clusters that formed a ring; intriguingly progression around the ring was associated with an increasing diurnal index from previous work. This is interesting, and discussed sensibly. It does bother me some that while this is true of 5 of the clusters, 8 of the clusters are NOT on the ring, and their diurnal score is not presented. These represent at least half the total cells, I would guess - so are these cells NOT at some random diurnal position, but in some off-state? What's going on? This is not discussed that I can see. There does appear to be relevant information in Fig. 3C, but this is not explained in a way that helps me much.

RESPONSE: Thank you for asking us to look more closely at the data. We initially excluded the clusters that were not directly aligned along the UMAP (or t-SNE) circle, but we have gone back and determined that all clusters were, in fact, highly rhythmic with distinct diurnal phases, with the possible exception of clusters #12 and #14. The diurnal phase distribution in clusters 7-11 falls very nicely in between that of clusters 6 and 1, that is the clusters they are the closest neighbors to in the UMAP and t-SNE plots.

We also repeated the pseudo-time trajectory analysis with all clusters, which again placed clusters 6-11 in between clusters 0 and 2, which is in agreement with the heatmap results.

Last, we extended this analysis to the Fe– cells as well, with very much the same outcome: all cell clusters are rhythmic. We do note, however, that Fe-deficient cells appear to be less synchronized than their Fe sufficient controls, suggesting a role for Fe nutrition in entrainment of the circadian clock, as was shown in Arabidopsis (Salomé et al., EMBO J 2013).

We have modified Figures 3 and 4 accordingly, and adjusted the corresponding text in the Results section (Lines 339-370).

Point 5. I'm sorry to say that I really don't understand the 'pseudo-time' assignment. It seems to me redundant with the diurnal assignment of clusters already carried out. Somewhat as with UMAP, it appears that you can only understand this analysis by knowing something about a program called Monocle. I never heard of it, and unless everyone else in the TPC readership is like 'Of course, Monocle, what else would you use?', I think the authors of the present MS ought to explain what this program does and why it is appropriate.

RESPONSE: Pseudo-time analysis determines the trajectory of a continuous process that describes the transcriptional changes between cells and then arranges cells based on their position along this trajectory. These trajectories may either describe shorter term temporal changes, or longer term developmental ones. Because we suspected that our cells were differing based on their diurnal cycle, the use of pseudo-time to order them along a trajectory seemed like a natural way to validate our hypothesis and treat cellular states as continuous rather than discrete (as we had done in the previous cluster based analysis).

To make the text more logical in its progression, we now introduce Monocle and its application to our data BEFORE moving to the diurnal phase part of the results. This felt more intuitive during the revision of the manuscript, and also helps us assign a hierarchy of progression from one cluster to the next which greatly helps the interpretation of the diurnal data later (Lines 363-370).

Moreover, in the prior clustering analysis, we considered each cluster as a unit, which ignores the heterogeneity of the cells inside a cluster. When using pseudo-time analysis, the cells are aligned linearly along the deduced time trajectory, which allows us to view the continuum of diurnal states across the entire population of cells rather than an average (Lines 409-415). Monocle is one widely used pseudo-time trajectory inference method. We think other pseudo-time analysis methods would also work on this dataset as the progression pattern is so obvious.

Point 6. The paper shows that the methodology works with cells with or without cell walls, which is technically a helpful thing, and further, that cell-wall-less strains have their own (UMAP!) gene signature.

RESPONSE: Thank you for this comment.

The separation between the cells with or without cell walls on the UMAP may reflect differences in gene expression between the two strains. It is also possible that some transcripts are preferentially extracted or protected as a consequence of the physical barrier (or lack thereof) that the cell wall constitutes in the *CW* strain. One caveat is that the strains used here are not isogenic, so any numbers of polymorphisms may contribute to the observed pattern.

Therefore, we believe that the expression difference is inherent to the cells and not specific to any dimensionality reduction methods, UMAP was only used to make the difference visible. We have bulk RNA-seq data on multiple strains, and it will be informative to compare the complement of genes detected by each method.

Overall, the authors have done a useful thing by validating scRNA in Chlamydomonas, and I am generally convinced by their controls. The diurnal/random phase in continuous light idea is interesting but seemingly incomplete given that 8/13 clusters don't fit the picture (or else I just misunderstand). In general, for a paper whose primary purpose is technical evaluation of a method, technical aspects are under-explored - prime examples are the degree and

consequences of undersampling, and the use of powerful computer programs that do things to the data that the reader (at least this reader) can't figure out to produce visually appealing but less-than-fully-informative images.

RESPONSE: Thank you!

Reviewer #3:

The manuscript described the scRNA-seq method in the model green alga *Chlamydomonas reinhardtii*. The authors performed scRNA-seq analysis in Chlamydomonas under iron- and nitrogen-deficient conditions and obtained results that were in good agreement with previous findings, demonstrating that the method works in Chlamydomonas. They also found that heterogeneity existed among individual cells under iron-replete and iron-deficient conditions and showed that this can be well explained by diurnal variation in gene expression. Finally, it is shown that the method can be applied to cells with and without cell walls.

The applicability of the scRNA-seq method to Chlamydomonas is well supported by the results obtained here. It is also interesting that the asynchronous cultures of Chlamydomonas contain cells of various diurnal phases. In general, conventional analysis using RNA from the entire population of asynchronous cultures (Northern blot, RT-qPCR) result in the loss of circadian rhythmicity during long-term cultivation without entrainment stimuli. This phenomenon is explained by the asynchrony among individual cells, as has been demonstrated by the analysis of specific reporter gene rhythms in other model systems. It has been believed that the situation is the same in Chlamydomonas. This study demonstrated this point clearly by global gene expression, rather than by a limited number of reporter genes. This is expected to have a significant impact on researchers in the field of chronobiology. Furthermore, the findings of this study will provide an important basis for future studies using scRNA-seq method in Chlamydomonas.

Major points

None

Minor points

The heterogeneity appears smaller under nitrogen-depleted conditions than under other conditions (Fe+, Fe-). It is also interesting that there is no cyclic distribution under nitrogen-depleted conditions. Does the diurnal rhythm disappear under nitrogen-deficient conditions? The authors might want to mention this point.

RESPONSE: As hinted at by reviewer 2, UMAP plots are not always informative, and we believe this might be such an example. While it is true that N– cells appear as a more condensed group in the UMAP plots shown in Figure 2, we do not believe that the size of the area they cover represents a quantitative measure of diversity.

N-deficient cells do appear to occupy a preferential diurnal phase around 10-12 h corresponding to the timing of cell division, which we interpret as a cell division block that prevents cells from dividing so as to maintain their N quota. A similar effect has been seen in Chlamydomonas cultures treated with cycloheximide (Howell, Blaschko and Drew, 1975, JCB, https://www.ncbi.nlm.nih.gov/pmc/articles/PMC2109581/pdf/jc671126.pdf). This study is now cited.

line 249: This sentence seems to be a description of S Fig 1B, not S Fig 2B.

RESPONSE: Fixed, thank you!

TPC2020-RA-00762R1 2nd Editorial decision – *acceptance pending* **January 3, 2021**

We are pleased to inform you that your paper entitled "Single-Cell RNA Sequencing of Batch Chlamydomonas Cultures Reveals Heterogeneity in their Diurnal Cycle Phase" has been accepted for publication in The Plant Cell, pending a final minor editorial review by journal staff.

Final acceptance from Science Editor **January 13, 2021**
